# AFAP1L1 promotes gastric cancer progression by interacting with VAV2 to facilitate CDC42-mediated activation of ITGA5 signaling pathway

**DOI:** 10.1186/s12967-023-03871-8

**Published:** 2023-01-11

**Authors:** Bo Sun, Bai Ding, Yu Chen, Chuang Peng, Xu Chen

**Affiliations:** Department of Hepatobiliary Surgery, Hunan Provincial People’s Hospital, The First Affiliated Hospital of Hunan Normal University, No. 61 Jiefang West Road, Changsha, 410005 Hunan China

**Keywords:** Actin filament associated protein 1 like 1, Epithelial-mesenchymal transition, Gastric cancer, Integrin subunit alpha 5

## Abstract

**Background:**

The actin filament-associated protein (AFAP) family genes include AFAP1/AFAP-110, AFAP1L1 and AFAP1L2/XB130. Increasing evidence indicates these three AFAP family members participate in tumor progression, but their clinical significance and molecular mechanisms in gastric cancer (GC) remain unclear.

**Methods:**

We first analyzed expression of AFAP family genes using public datasets and verified the results. The clinical significance of AFAP family genes in GC patients was also analyzed. In vitro and in vivo experiments were applied to explore the function of AFAP1L1. Enrichment analysis was used to explore potential molecular mechanisms. We then performed additional experiments, such as cell adhesion assay, co-immunoprecipitation and so on to confirm the downstream molecular mechanisms of AFAP1L1.

**Results:**

Public data analyses and our verification both showed AFAP1L1 was the only AFAP family members that was significantly upregulated in GC compared with normal gastric tissues. Besides, only AFAP1L1 could predict poor prognosis and act as an independent risk factor for GC patients. In addition, AFAP1L1 promotes GC cells proliferation, migration, invasion in vitro and tumor growth, metastasis in vivo by inducing epithelial-to-mesenchymal transition (EMT). In terms of mechanism, AFAP1L1 interacts with VAV guanine nucleotide exchange factor 2 (VAV2) to activate Rho family GTPases CDC42, which finally promotes expression of integrin subunit alpha 5 (ITGA5) and activation of integrin signaling pathway.

**Conclusion:**

AFAP1L1 promotes GC progression by inducing EMT through VAV2-mediated activation of CDC42 and ITGA5 signaling pathway, indicating AFAP1L1 may be a promising prognostic biomarker and therapeutic target for GC patients.

**Supplementary Information:**

The online version contains supplementary material available at 10.1186/s12967-023-03871-8.

## Background

Recent epidemiological study shows that gastric cancer (GC) is an important lethal malignant tumor worldwide and ranks fifth for incidence and fourth for mortality globally [1]. Although many new chemotherapies and surgical technologies have improved the prognosis of GC patients, the 5-year survival rate is still poor at about 30%, for most GC cases are detected at advanced stages [2]. Tumor metastasis is associated with chemoresistance and recurrence, which is the major reason for restricting the improvement of prognosis in GC patients. Invasion-metastasis cascade is a complex biological process, involving molecular, cellular and organic regulatory mechanism [3]. Therefore, elucidating molecular mechanisms of GC invasion-metastasis cascade will contribute to discover new targeted treatment and further improve prognosis of GC patients.

The actin filament-associated protein (AFAP) family is a kind of adaptor protein, including AFAP1/AFAP-110, AFAP1L1 and AFAP1L2/XB130. Adaptor proteins often lack enzymatic activity and interact with other proteins to form various signal complexes, which could activate downstream signaling pathways through specific protein–protein interactions [4]. The three members of AFAP family could bind actin filament and cSrc-activating protein due to their similar modular domain structure and amino acid sequence, particularly for their highly conserved PH domain and SH2/SH3 binding domain motifs [5, 6]. However, the existing differences in structure also lead to different subcellular localization and determine their binding molecules which are involved in diverse signaling pathways with significantly different cellular functions.

At present, some studies have found AFAP family members could affect tumor cell proliferation, invasion, epithelial-mesenchymal transition (EMT) and participate in tumor progression. AFAP-110 is upregulated in prostate cancer and is associated with prostate cancer progression [7]. AFAP1L1 promotes cell proliferation, cell cycle progression and inhibits cell apoptosis in non-small cell lung cancer [8]. Besides, AFAP1L1 also acts as oncogene in colorectal cancer and spindle cell sarcomas [9, 10]. Although AFAP1L2 is also associated with tumor progression, such as prostate cancer [11], non-small cell lung cancer [12] and breast cancer [13], AFAP1L2 acts as a tumor suppressor in carcinogen-induced skin tumorigenesis [14]. EMT, a cellular trans-differentiation process that involves a dramatic reorganization of the actin cytoskeleton, is considered as an important factor for cancer cell migration, invasion and metastasis [15]. However, the present studies report that only AFAP1L2 of these three members is associated with EMT [12, 13, 16]. Despite these intriguing studies, the functional role, relationship with EMT, and clinical significance of AFAP family members in GC are still elusive. Systematic study of AFAP family members in GC will help us to understand the molecular basis for GC development and find potential therapeutic targets.

In this study, we found AFAP1L1 was the only AFAP family members that was significantly upregulated in GC compared with normal gastric tissues. What’s more, only AFAP1L1 was associated with poor prognosis and was an independent risk factor for overall survival in GC patients. In vitro and in vivo experiments demonstrated that AFAP1L1 promoted GC cells proliferation, invasion, metastasis and EMT process. In terms of mechanism, AFAP1L1 interacted with VAV guanine nucleotide exchange factor 2 (VAV2) to activate CDC42, a small GTPase of the Rho-subfamily, which finally promoted expression of integrin subunit alpha 5 (ITGA5) and activation of integrin signaling pathway.

## Methods

### Bioinformatics analysis

The mRNA expression data and overall survival data of AFAP1, AFAP1L1 and AFAP1L2 in The Cancer Genome Atlas (TCGA) STAD database were downloaded from the UCSC Xena browser (http://xena.ucsc.edu/) [17]. GSE27342 [18] in the Gene Expression Omnibus (GEO) database was used to analyze mRNA expression of AFAP family members. We also analyzed the effect of AFAP family members on survival of GC patients in Kaplan-Meier Plotter website (http://kmplot.com/analysis/) [19]. The association between AFAP family members and clinicopathological characteristics and uni-/multi-variate analyses used to identify independent risk factors were both analyzed in Xiantao academic online analysis tool (https://www.xiantao.love/) using TCGA STAD database (https://portal.gdc.cancer.gov/). Gene Set Enrichment Analysis (GSEA) method was adopted to conduct Gene Ontology (GO) and Kyoto Encyclopedia of Genes and Genomes (KEGG) pathway analyses using TCGA STAD database. GSEA was performed using the clusterProfiler R package with false discovery rate (FDR) < 0.25 and *P* < 0.05 as the cut-off criteria.

### Patients and specimens

A total of 40 pairs of randomly selected snap-frozen GC tissues and adjacent nontumorous gastric tissues (ANGTs) from initial surgical resection were obtained from Hunan Provincial People’s Hospital/The First Affiliated Hospital of Hunan Normal University and were used to qRT-PCR and western blot analysis. Meanwhile, these fresh GC tissues were fixed with formalin and paraffin embedded and further used to immunohistochemistry analysis. All patients were diagnosed with GC by two independent pathologists.

### RNA extraction and qRT-PCR

TRIzol™ Reagent (ThermoFisher Scientific, Waltham, MA) was used to isolate total RNA according to manufacturer’s protocol. cDNA was synthesized using the ReverTra Ace® qPCR RT Master Mix (Toyobo, Japan) according to manufacturer’s instructions. qRT-PCR was performed using the SYBR® Green Realtime PCR Master Mix-Plus kit (Toyobo, Japan) in QuantStudio3 System (Applied Biosystems, CA). GAPDH was used as internal control. The mRNA expression level was calculated by 2^−ΔCt^ or 2^−ΔΔCT^ method based on cycle threshold (Ct). The experiments were done in triplicates. The primer sequences used in this study were listed in Additional file 1: Table S1.

### Protein extraction and western blot

Total protein was extracted by RIPA lysis buffer (NCM, Soochow, China) containing Protease and Phosphatase Inhibitor Cocktail (NCM, Soochow, China). Equal amounts of total protein sample were separated by SDS-PAGE gel (NCM, Soochow, China) and further transferred to PVDF membrane (Millipore, Bedford, MA). The membranes were incubated with appropriate primary antibodies and then HRP conjugated secondary antibodies. β-actin was used as loading control. All the primary antibodies used in this study were listed in Additional file 1: Table S2.

### Immunohistochemistry (IHC)

IHC assay was conducted using streptavidin-peroxidase method (ZSGB-BIO, Beijing, China). In brief, clinical sample sections (4 μm) were firstly dewaxed, hydrated and antigen retrieved. After inactivating endogenous peroxidase, the sections were blocked with normal goat serum and incubated with appropriate primary antibodies overnight at 4 °C. Then, the sections were sequentially incubated with reaction enhancement solution, secondary antibodies solution and DAB peroxidase substrate. Finally, the sections were stained by hematoxylin, and further dehydrated and mounted. The primary antibodies used for IHC are listed in Additional file 1: Table S2. IHC score was calculated according to proportion and intensity of positive cells [20, 21], namely IHC score = intensity score × percentage score. The score of staining intensity: 0, negative; 1, weak; 2, moderate; 3, strong. The score of positive cells: 0, less than 5% positive cells; 1, 5 ~ 25% positive cells; 2, 26 ~ 50% positive cells; 3, 51 ~ 75% positive cells; 4, > 75% positive cells.

### Cell lines and cell culture

Human gastric mucosal epithelial cell line GES-1 and five GC cell lines (AGS, MKN74, MGC-803, HGC27 and MKN45) were purchased from the Type Culture Collection of the Chinese Academy of Sciences (China) and maintained in RPMI-1640 medium (BioInd, Beit Haemek, Israel) supplemented with 10% FBS (BioInd, Beit Haemek, Israel) and 1% penicillin/streptomycin (BioInd, Beit Haemek, Israel) in humidified incubator at 37 °C with 5% CO_2_. Short tandem repeat (STR) analysis was used to authenticate all cell lines before experiments and all mycoplasma test were negative.

### Vector construction and transfection

Lentiviruses containing AFAP1L1/VAV2/ITGA5 short hairpin RNAs (shRNA) or their open reading frame (ORF) and corresponding negative control lentiviruses were purchased from WZ Biosciences (Shandong, China). Transfection was performed according to manufacturer’s instructions. The transfection of lentiviruses was performed using Lipofectamine 2000 (Invitrogen, Carlsbad, CA). Puromycin (2 µg/mL) was used to select stable clones. The knockdown and overexpression efficacy of corresponding lentiviruses were verified by qRT-PCR and western blot. The sequence of VAV2 shRNA was as follow: 5′-GCATGACTGAAGATGACAAGA-3′. The sequence of ITGA5 shRNA was as follow: 5′-GCAGAGAGATGAAGATCTACC-3′. The sequences of AFAP1L1 shRNA were as follows: shRNA1: 5′-GGGCGCAACTCCTTCCTATAT-3′; shRNA2: 5′-GGTGTGGGTGACAACTGTTCT-3′; shRNA3: 5′-GCAAGTCGCCTGAGTACATCA-3′.

### In vitro cell proliferation assays

CCK8 assay, colony formation assay and Edu assay were performed to test the role of AFAP1L1 on GC cell proliferation. For CCK8 assay, around 2 × 10^3^ cells were seeded into 96-well plates. After cell attachment, the cells were incubated with CCK8 solution for 2 h and subjected to determine absorbance at 450 nm using spectrophotometer. For colony formation assay, around 5 × 10^2^ GC cells were added into 6-well plates and cultured in incubator for 2 weeks. Then, the colonies were stained by crystal violet (Beyotime Biotechnology, Shanghai, China) and counted for diameter more than 40 μm. The Edu assays were performed using Cell-Light EdU Apollo567 In Vitro Kit (RiboBio, Guangzhou, China) according to the manufacturer’s protocol. The percentage of Edu positive cells was calculated and compared. All experiments were repeated three times.

### Immunofluorescence

GC cells were seeded into 6-well plates inserted with glass coverslip. After attachment, cells in the coverslip were fixed by 4% formaldehyde and perforated by 0.5% Triton X-100. Then, the cells were incubated with primary antibody overnight at 4 °C. Appropriate fluorescence labeled secondary antibodies were used to detect corresponding proteins. In addition, rhodamine-conjugated phalloidin (Roche, Basel, Switzerland) was used to detect cytoskeleton of GC cells. Nuclei were stained by DAPI solution (Beyotime Biotechnology, Shanghai, China). The primary antibodies for immunofluorescence used in this study were listed in Additional file 1: Table S2.

### Transwell migration and invasion assays

For transwell migration assay, the suspended GC cells were added into upper chamber of transwell insert (Corning, Kennebunk, ME) without Matrigel and cultured for 24 h. For transwell invasion assay, GC cells were added into upper chamber of the transwell insert (Corning, Kennebunk, ME) with Matrigel (BD Biosciences, Franklin Lakes, NJ) and cultured for 24 h. The cells that invaded to bottom membrane of chamber were fixed and stained with 0.1% crystal violet (Beyotime Biotechnology, Shanghai, China). Mitomycin-C (10 µg/mL, Sigma, St. Louis, MO) was used to suppress cell proliferation before the experiments. All experiments were repeated three times.

### HCC mouse model and in vivo study

All in vivo studies were performed using 6-week-old male BALB/c nude mice. The subcutaneous mouse model was used to detect GC cell growth in vivo. In short, around 5 × 10^6^ cells within 200 μL cool PBS were injected subcutaneously into left upper flank region of nude mice. The subcutaneous tumor volume was calculated after 6 weeks using Vernier caliper as follow: tumor volume (mm^3^) = (L × W^2^)/2, where L = long axis and W = short axis. For in vivo metastatic assay, nude mice were injected with 1 × 10^6^ GC cells (resuspended in PBS) via tail vein. After four weeks, all the lungs and livers were fixed with formalin and sectioned serially and stained with hematoxylin and eosin (H&E) for histological examination. The number of metastatic nodules in mice lung and liver was analyzed and compared.

### Cell adhesion assay

About 1 × 10^4^ suspended GC cells were placed into each well of a collagen-coated 96-well plate and cultured at 37 °C for 1 h. Then, cells that did not adhere to the plate were washed off with PBS. The remaining GC cells bound to collagen on plate were stained by SRB Assay Kit (Abcam, Cambridge, England) according to the manufacturer’s protocol. The final absorbance was measured at 565 nm. Plates incubated with bovine serum albumin (BSA) were used as negative control. All experiments were repeated three times.

### Co-immunoprecipitation (co-IP)

Co-IP was performed using a Pierce™ Classic Magnetic IP/Co-IP Kit (ThermoFisher Scientific, Waltham, MA) following manufacturer’s protocol. In brief, monolayer GC cells were lysed and centrifuged and the protein concentration was tested. Then, a total of 1000 μg proteins were incubated with 10 µg of IP antibody (AFAP1L1 or VAV2) or control IgG at 4 °C overnight to form the immune complex. After washing magnetic beads using IP Lysis/Wash Buffer, the protein sample/antibody immune complex was added into the washed magnetic beads and incubated at room temperature for 1 h with mixing. Finally, the target antigen was disassociated from the beads using Elution Buffer and the samples were analyzed by western blot.

### Pull-down assays

The level of active CDC42 (GTP-CDC42) and active Rac1 (GTP-Rac1) in GC cells was measured using Rac1/Cdc42 Activation Assay Kit (Millipore, MA) according to the manufacturer’s protocol. Briefly, GC cells were lysed by lysis buffer. PAK1-PBD agarose beads, which specifically bound to active CDC42 and Rac1, were added into the cell lysates. Agarose beads were then washed with lysis buffer three times. Active CDC42 and Rac1 was disassociated from PAK1-PBD agarose beads by reduced sample buffer and boiled for 5 min. The final samples were analyzed by western blot using anti-CDC42 antibody or anti-Rac1 antibody. Similarly, the level of active RhoA (GTP-RhoA) in GC cells was measured by RhoA Pull-down Activation Assay Biochem Kit (Cytoskeleton, CO) using Rhotekin-RBD bead according to the manufacturer’s protocol.

### Statistical analysis

Statistical analyses were performed using SPSS 20.0 (SPSS Inc., Chicago, IL) or Graphpad Prism 8 (GraphPad Software, San Diego, CA). The differences between two groups were analyzed by Student’s *t* test when the variance is homogeneous or Mann-Whitney *U* test when the variance is inhomogeneous. Categorical data was analyzed using Chi-square test or Fisher exact test. Correlation coefficient was determined by Pearson correlation analysis. Survival curves were depicted by Kaplan-Meier method and compared by log-rank test. Univariate and multivariate analyses were performed to determine prognostic factors based on Cox proportional hazards regression model. Data were expressed as mean ± SD from at least three independent experiments. *P* < 0.05 (two-tailed) was considered statistically significant.

## Results

### AFAP1L1 is significantly upregulated in GC tissues and cell lines

**Fig. 1:**
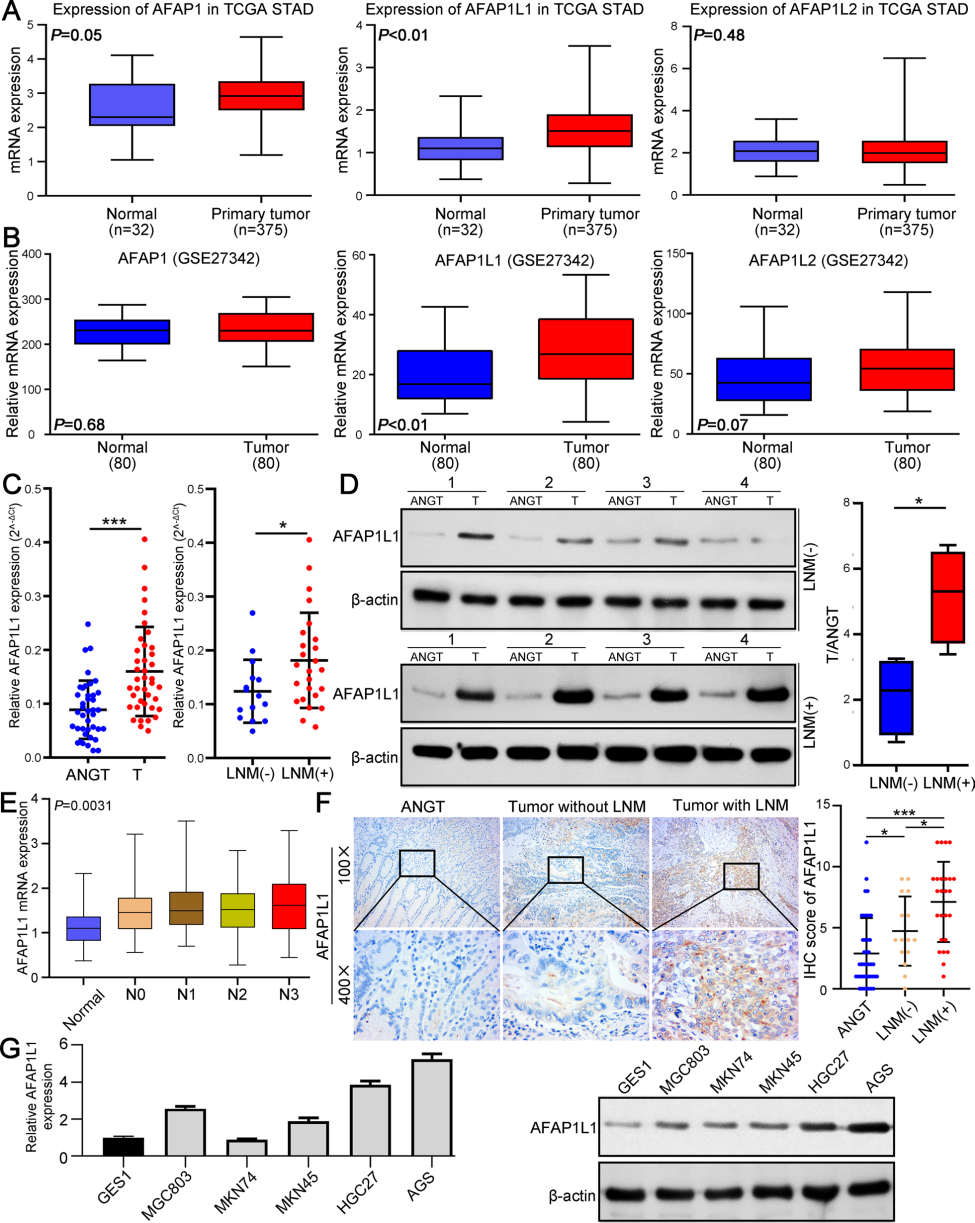
AFAP1L1 is significantly upregulated in GC tissues and cell lines. **A** The expression of AFAP family members in GC tissues and normal gastric tissues was analyzed using TCGA STAD data. **B** Comparing the expression level of AFAP family members in GC tissues and normal gastric tissues using GES27342 database. **C** qRT-PCR analysis of AFAP1L1 mRNA expression in 40 pairs of randomly selected snap-frozen GC tissues and ANGTs. The mRNA expression of AFAP1L1 in GC tissues with or without LNM was further compared. **D** Western blot analysis of AFAP1L1 protein expression in representative ANGTs and GC tissues with LNM (lower panel) or without LNM (upper panel). The results were compared in the right boxplot. **E** The expression of AFAP1L1 in TCGA STAD database stratified by the number of LNM. **F** Representative IHC staining for AFAP1L1 in ANGT, GC without LNM and GC with LNM (left panel). IHC staining score of AFAP1L1 in ANGT, GC without LNM and GC with LNM was compared (right panel). **G** The mRNA and protein expression of AFAP1L1 in gastric mucosal epithelial cell line GES-1 and five GC cell lines. ANGT: adjacent nontumorous gastric tissue; T: gastric cancer tissue; LNM: lymph node metastasis. *P < 0.05; ***P < 0.001

To explore the mRNA expression of AFAP1L1 family members in gastric normal tissues and GC tissues, we first analyzed TCGA STAD database (Fig. 1A) and GSE27342 in GEO database (Fig. 1B) and the results both showed AFAP1L1 was the only AFAP family member that was significantly upregulated in GC tissues. Then, we randomly selected 40 pairs fresh GC tissues and corresponding ANGTs and performed qRT–PCR to analyze expression of AFAP family members. The results also showed AFAP1L1 was the only one upregulated in GC tissues (Fig. 1C, Additional file 2: Fig. S1A). More importantly, GC with lymph node metastasis (LNM) had significantly higher AFAP1L1 expression than those without LNM (Fig. 1C). Western blot of representative samples further confirmed upregulation of AFAP1L1 protein in GC and its higher expression in GC with LNM (Fig. 1D). We then analyzed expression of AFAP family members in TCGA STAD database stratified by LNM status. Only AFAP1L1 expression was gradually elevated as the LNM number increases (Fig. 1E, Additional file 2: Fig. S1B). IHC score of AFAP1L1 also increased progressively from ANGTs, GC without LNM, to GC with LNM (Fig. 1F; Additional file 2: Fig. S1C). In addition, most GC cell lines displayed higher mRNA and protein expression of AFAP1L1 than gastric mucosal epithelial cell line GES-1 (Fig. 1G). In summary, AFAP1L1 expression is upregulated in GC tissues and may be associated with GC metastasis.

### AFAP1L1 is associated with poor prognosis of GC patients

**Fig. 2:**
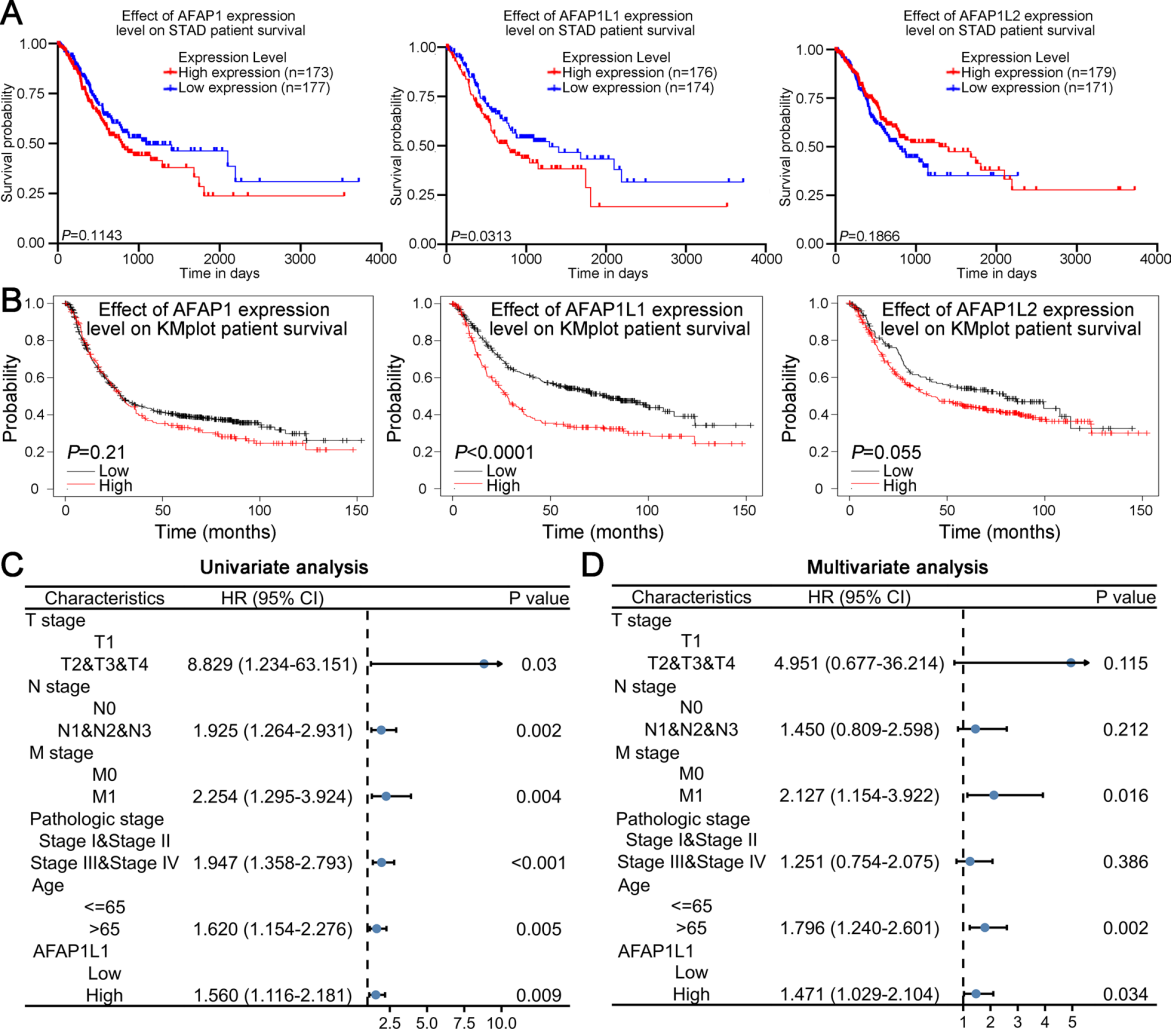
AFAP1L1 is associated with poor prognosis of GC patients. **A** Kaplan–Meier analysis of overall survival for GC patients based on AFAP1L1 mRNA expression using follow-up data from the TCGA STAD database. **B** Kaplan–Meier analysis of overall survival for GC patients based on AFAP1L1 mRNA expression using follow-up data from the Kaplan–Meier Plotter website. **C** Forest plot by data from univariate analysis using TCGA STAD database depicted hazard ratio of overall survival for indicated clinical subgroups. **D** Forest plot by data from multivariate analysis using TCGA STAD database depicted hazard ratio of overall survival for indicated clinical subgroups. HR: hazard ratio

We then analyzed the association between expression of AFAP family members and survival outcome using data from TCGA STAD database and Kaplan-Meier Plotter website. Intriguingly, the two analytical methods both showed that GC patients with high AFAP1L1 expression had significantly worse prognosis than those with low AFAP1L1 expression, but the expression of AFAP1 and AFAP1L2 had no influence on survival of GC patients (Fig. 2A, B). Besides, the association between expression of AFAP family members and clinicopathological characteristics in TCGA STAD database was also analyzed. Clinicopathological characteristics were several factors that had been systematically studied in GC and were associated with prognosis of GC patients. For example, GC patients with diffuse type cancer, tumor in fundus/body, advanced TNM stage or advanced pathologic stage often exhibited poorer prognosis compared to those with contrary characteristics. Patients with different clinicopathological characteristics may have different molecular subtypes. Analysis of the association between AFAP family members and clinicopathological characteristics in GC patients could help us to better understand the biological functions of AFAP family in GC progression. Our analysis showed AFAP1 was significantly associated with histological type (*P* = 0.024) and anatomic neoplasm subdivision (*P* = 0.014) (Additional file 1: Table S3). AFAP1L1 was significantly associated with T stage (*P* = 0.014) and histological type (*P* = 0.024) (Additional file 1: Table S4). There was no clinicopathological characteristics that had significant association with AFAP1L2 expression in GC (Additional file 1: Table S5). Then, univariate analysis indicated that T stage, N stage, M stage, Pathologic stage, Age and AFAP1L1 expression were risk factors for overall survival (Fig. 2C, Additional file 1: Table S6). Multivariate analysis further showed that M stage, age and AFAP1L1 expression were independent risk factors for overall survival (Fig. 2D, Additional file 1: Table S6). Collectively, AFAP1L1 is the unique AFAP family members that could predict prognosis of GC patients and could serve as an independent prognostic biomarker.

### High AFAP1L1 expression in GC promotes proliferation, invasion in vitro and growth, metastasis in vivo

**Fig. 3:**
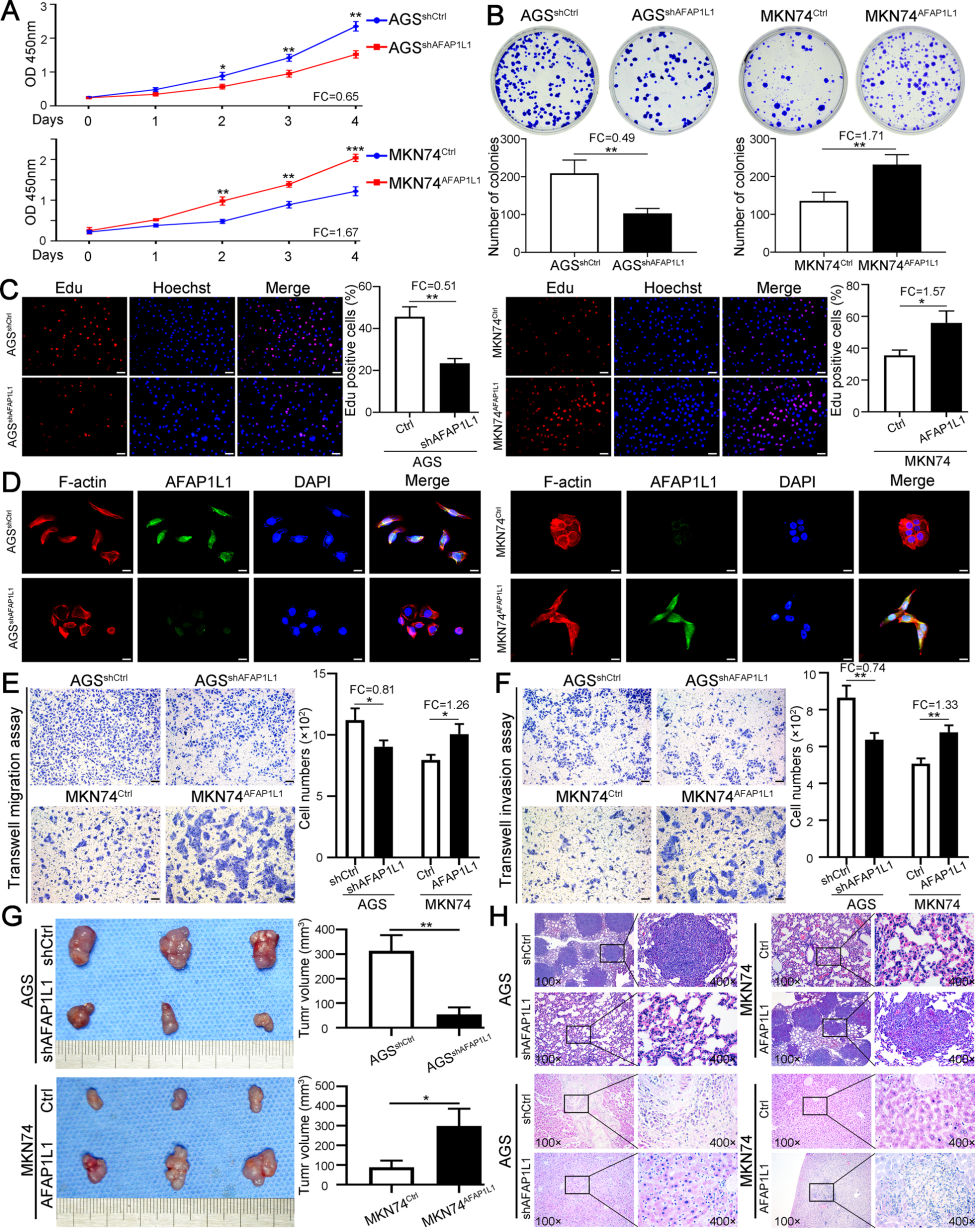
High AFAP1L1 expression in GC promotes proliferation, invasion in vitro and growth, metastasis in vivo. **A** The cell proliferative curves were plotted by CCK8 assays for AGS^shAFAP1L1^, MKN74^AFAP1L1^ and their control cells. **B** The representative images and the quantification of colony formation assays from AGS^shAFAP1L1^, MKN74^AFAP1L1^ and their control cells. **C** Edu assays showed the proliferating cells in AGS^shAFAP1L1^, MKN74^AFAP1L1^ and their control groups. The percentage of proliferating cells was compared in corresponding bar graphs. Scale bars, 50 μm. **D** Double fluorescent staining of cytoskeleton (red) and AFAP1L1 (green) for indicated GC cells. Cytoskeleton was stained by rhodamine phalloidin and Nuclei was stained with DAPI (blue). Scale bars, 10 μm. **E**, **F** The influence of AFAP1L1 overexpression or knockdown on GC cells migratory and invasive capacity was determined by transwell migration (**E**) and invasion assays (**F**). Scale bars, 50 μm. **G** Subcutaneous tumor model was constructed to test the effect of AFAP1L1 overexpression or knockdown on GC cells growth in vivo. The final tumor volumes were compared in right bar graphs. **H** In vivo metastatic assays by tail vein injection of indicated GC cells and representative H&E staining images of pulmonary and intrahepatic metastases. FC: fold change. *P < 0.05; **P < 0.01; ***P < 0.001

To determine the cellular function of AFAP1L1 in GC, we first selected AGS cell line with high AFAP1L1 expression to transfect with lentiviruses containing shRNAs for AFAP1L1 and MKN74 cell line with lower AFAP1L1 expression to overexpress AFAP1L1 by transfecting lentiviruses containing ORF of AFAP1L1. The knockdown and overexpression efficacy were confirmed by qRT-PCR and western blot (Additional file 2: Fig. S2A, S2B). We then performed CCK8 assay, colony formation assay and Edu assay to test the role of AFAP1L1 on GC cells proliferation. All the experiments showed that AFAP1L1 knockdown in AGS could inhibit its proliferation, while overexpression of AFAP1L1 in MKN74 significantly promoted its proliferative capacity. The fold changes of CCK8 assay, colony formation assay and Edu assay after AFAP1L1 knockdown in AGS were 0.65, 0.49 and 0.51 respectively, while the fold changes of CCK8 assay, colony formation assay and Edu assay after AFAP1L1 overexpression in MKN74 were 1.67, 1.71 and 1.57 respectively (Fig. 3A–C). AFAP1L1 is a kind of actin filament-associated protein and may regulate cytoskeleton remodeling. Cytoskeletal staining by phalloidin revealed that knockdown of AFAP1L1 in AGS cells could make elongated cytoskeleton transform to ovoid morphology, while AFAP1L1 overexpression in MKN74 caused opposite transformation. Besides, we could see colocalization of AFAP1L1 with cytoskeleton, indicating AFAP1L1 participated in rearrangement of elongational cytoskeleton (Fig. 3D, Additional file 2: Fig. S2C). Transwell migration and invasion assays both showed that AFAP1L1 downregulation significantly decreased migratory (fold changes = 0.81) and invasive (fold changes = 0.74) capacity of AGS cells, while AFAP1L1 overexpression significantly increased migration (fold changes = 1.26) and invasion (fold changes = 1.33) of MKN74 cells (Fig. 3E, F). In vivo subcutaneous mouse model further demonstrated that AFAP1L1 downregulation inhibited growth of subcutaneous tumor derived from AGS cells, while AFAP1L1 overexpression obviously promoted tumor growth of MKN74 (Fig. 3G). Furthermore, in vivo metastatic experiments showed knockdown of AFAP1L1 in AGS cells significantly inhibited formation of pulmonary and liver metastasis focuses. MKN74 cells with AFAP1L1 overexpression metastasized more easily to lung and liver than control cells (Fig. 3H, Additional file 2: Fig. S2D). In a word, AFAP1L1 plays a critical role in promoting GC cells proliferation, invasion and metastasis.

### AFAP1L1 facilitates EMT of GC cells

**Fig. 4:**
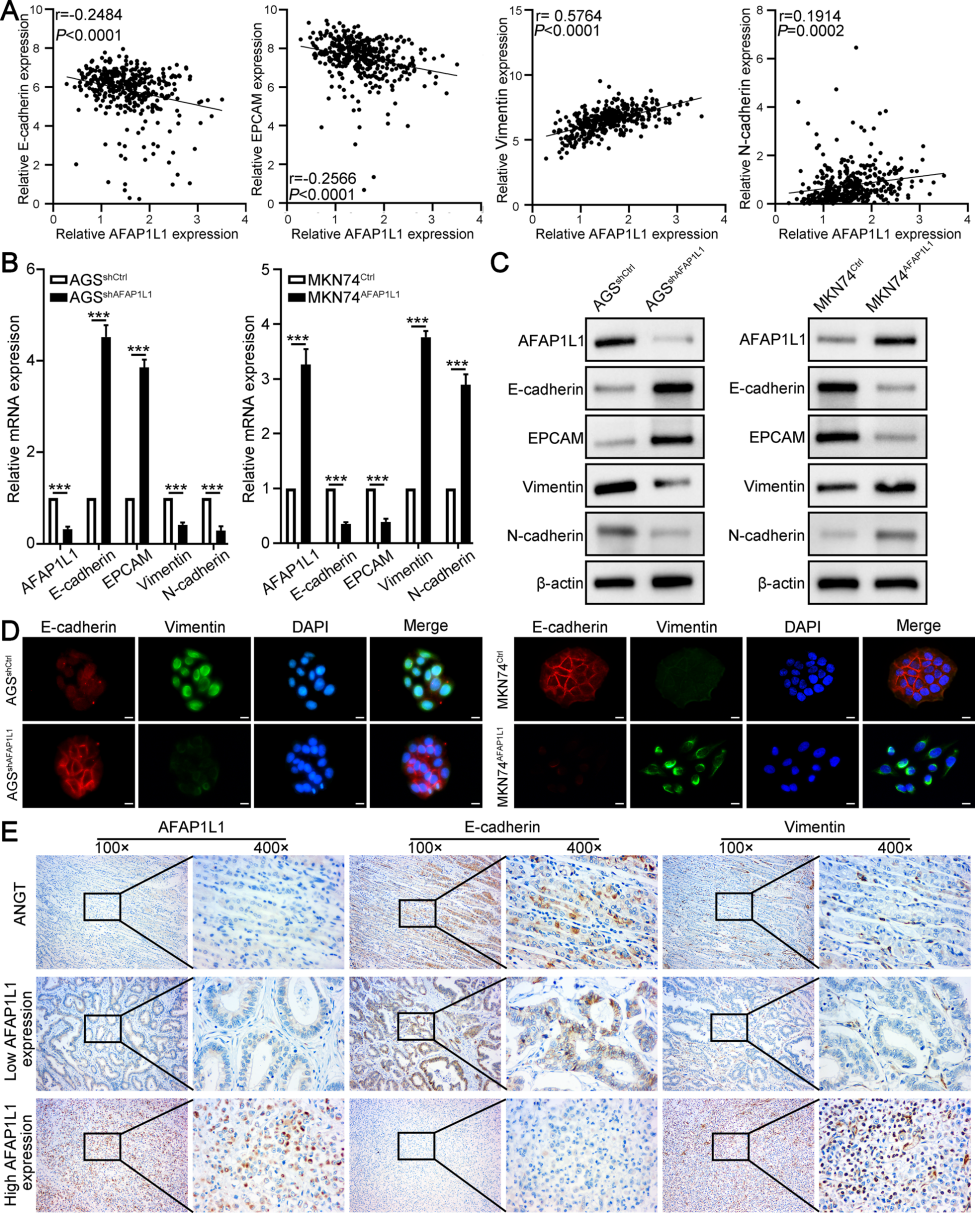
AFAP1L1 facilitates EMT of GC cells. **A** Pearson correlation analysis of AFAP1L1 and EMT markers (epithelial marker: E-cadherin, EPCAM; mesenchymal markers: vimentin, N-cadherin) in GC using data from TCGA STAD database. **B** qRT-PCR analysis of AFAP1L1 and EMT markers in AGS^shAFAP1L1^, MKN74^AFAP1L1^ and their control cells. **C** Western blot analysis of AFAP1L1 and EMT markers in AGS^shAFAP1L1^, MKN74^AFAP1L1^ and their control cells. **D** Double immunofluorescence staining of E-cadherin (red) and vimentin (green) in AGS^shAFAP1L1^, MKN74^AFAP1L1^ and their control cells. Nuclei was stained with DAPI (blue). Scale bars, 10 μm. **E** The representative IHC images from serial sections of GC samples showed AFAP1L1, E-cadherin and vimentin expression in ANGT and GC tissues with high or low AFAP1L1 expression. ***P < 0.001

The occurrence of EMT is often accompanied with reorganization of cytoskeleton which is necessary to destroy cell–cell attachment and strengthen cell–matrix adhesions [22]. AFAP1L1 could regulate cytoskeleton rearrangement, indicating AFAP1L1 may be also associated with EMT like AFAP1L2. We first analyzed correlation between AFAP1L1 and EMT markers in TCGA STAD database. Pearson correlation analysis revealed that AFAP1L1 was negatively correlated with epithelial markers (E-cadherin, EPCAM) and positively correlated with mesenchymal markers (vimentin, N-cadherin), strongly suggesting AFAP1L1 is associated with EMT of GC cells (Fig. 4A). We then tested mRNA and protein expression of these markers in above cell lines. The results showed AFAP1L1 downregulation in AGS cells could promote E-cadherin, EPCAM expression and inhibit vimentin, N-cadherin expression, whereas AFAP1L1 overexpression reduced expression of epithelial markers and increased expression of mesenchymal markers in MKN74 cells (Fig. 4B, C). Double immunofluorescence staining for E-cadherin and vimentin also confirmed AFAP1L1 could inhibit E-cadherin expression and promote vimentin expression in GC cells (Fig. 4D). Finally, we further performed IHC for serial sections of GC tissues and the results showed ANGTs and GC tissues with low AFAP1L1 expression had higher E-cadherin expression and lower vimentin expression, and vice versa in GC tissues with high AFAP1L1 expression (Fig. 4E). After calculating IHC score of E-cadherin and vimentin, Pearson correlation analysis demonstrated AFAP1L1 was negatively correlated with E-cadherin (r = − 0.5657) (Additional file 2: Fig. S3A) and positively correlated with vimentin (r = 0.6313) (Additional file 2: Fig. S3B) in GC tissues. These results demonstrated that AFAP1L1 could promote EMT of GC cells.

### AFAP1L1 is associated with integrin signaling and promotes ITGA5 expression in GC

**Fig. 5:**
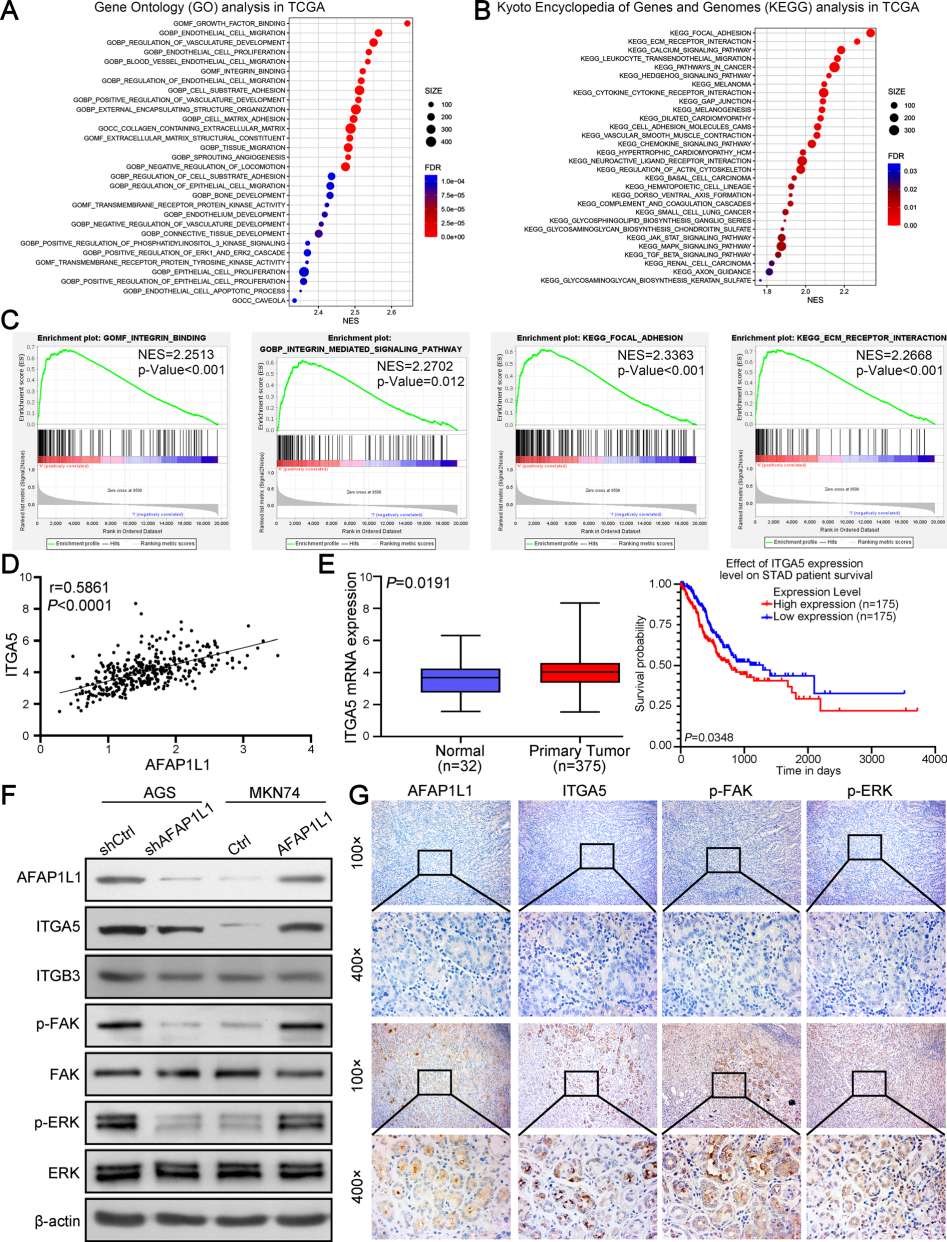
AFAP1L1 is associated with integrin signaling and promotes ITGA5 expression in GC. **A**, **B** GO enrichment analysis (**A**) and KEGG pathway analysis (**B**) were performed by differentially expressed genes (DEGs) between AFAP1L1 high- and low-expression group in TCGA STAD database. The top 30 pathways were shown in bubble diagrams. **C** Representative results of integrin-related pathways from Gene Set Enrichment Analysis (GSEA) of AFAP1L1 in TCGA STAD database. **D** Pearson correlation analysis of AFAP1L1 and ITGA5 in GC using data from TCGA STAD database. **E** Analysis using TCGA STAD database showed ITGA5 expression in GC tissues and normal gastric tissues and its effect on prognosis of GC patients. **F** Western blot analysis to detect the effect of AFAP1L1 overexpression or knockdown on the expression of ITGA5, ITGB3 and integrin downstream effectors FAK, ERK. p-FAK and p-ERK represented their activation forms. **G** The representative IHC images from serial sections of GC samples showed AFAP1L1, ITGA5, p-FAK and p-ERK expression in GC tissues with high or low AFAP1L1 expression

We then performed GO enrichment analysis and KEGG pathway analysis by differentially expressed genes (DEGs) between AFAP1L1 high- and low-expression groups in TCGA STAD database. GO enrichment analysis indicated that DEGs were mainly enriched in endothelial cell-related molecular functions (e.g. endothelial cell migration, proliferation and vasculature development) and integrin-related molecular functions (e.g. integrin binding, cell substrate adhesion and cell matrix adhesion) (Fig. 5A). Similarly, some integrin-related pathways were also enriched based on KEGG pathway analysis, including focal adhesion, ECM receptor interaction. Besides, signaling pathways associated with cancer progression, such as pathways in cancer, MAPK signaling pathway, were also enriched in KEGG pathway analysis (Fig. 5B) Gene set enrichment analysis (GSEA) using TCGA STAD data showed integrin was the mainly pathway enriched in high AFAP1L1 expression group (Fig. 5C). The integrin family comprises 24 transmembrane heterodimeric receptors generated by 18 α integrin and 8 β integrin subunits and acts as cell surface receptors for cell-ECM adhesion [23, 24]. We then analyzed the correlation between AFAP1L1 and 26 integrin subunits in TCGA STAD database (Additional file 2: Fig. S4A) and the results showed ITGA5 in α integrin subunit and ITGB3 in β integrin subunit were two integrin subunits that had the highest correlation coefficient (Fig. 5D, Additional file 2: Fig. S4B). However, when we further analyzed expression of ITGA5 and ITGB3 and their association with prognosis in GC patients, we found only ITGA5 was significantly upregulated in GC tissues and associated with poor prognosis of GC patients, indicating ITGA5 may be the functional mediator of AFAP1L1 in GC (Fig. 5E, Additional file 2: Fig. S4C, S4D). Indeed, knockdown of AFAP1L1 expression in AGS cells inhibited mRNA and protein expression of ITGA5, while AFAP1L1overexpression in MKN74 cells promoted ITGA5 mRNA and protein expression, but both had no influence on expression of ITGB3 (Fig. 5F, Additional file 2: Fig. S4E). Integrin-mediated cell proliferation, cell migration and cell adhesion generally require focal adhesion kinase (FAK) and MAPK/ERK signaling [25, 26]. We also found AFAP1L1 downregulation in AGS could inhibit phosphorylation of FAK and ERK, and AFAP1L1 overexpression in MKN74 could lead to the activation of FAK and ERK (Fig. 5F). Finally, IHC for serial GC sections also showed high AFAP1L1 co-expressed with ITGA5, p-FAK and p-ERK (Fig. 5G). In summary, AFAP1L1 could promote ITGA5 expression in GC and further activate its downstream effectors FAK and ERK.

### ITGA5 is necessary for AFAP1L1 mediated GC migration, invasion and EMT

**Fig. 6:**
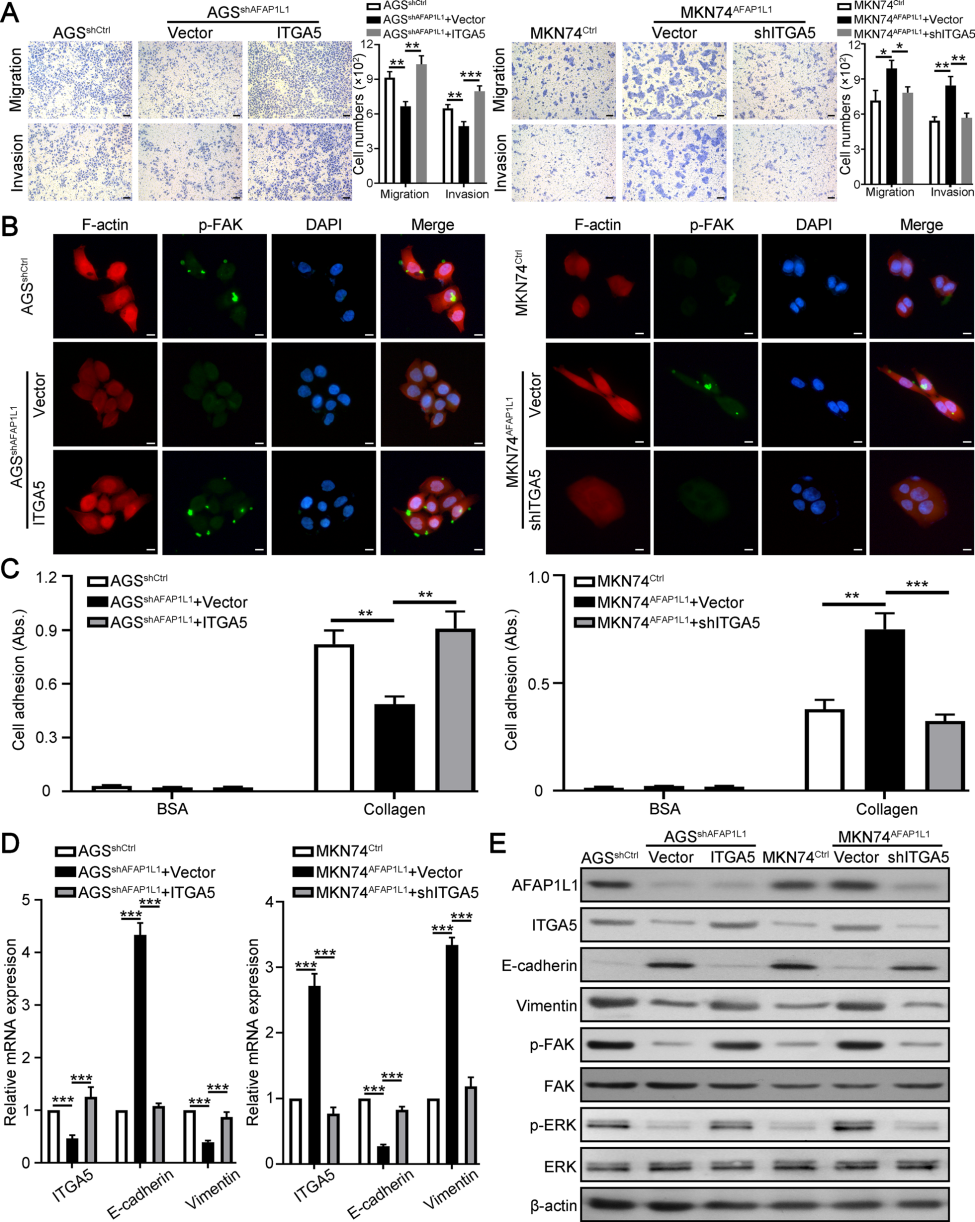
ITGA5 is necessary for AFAP1L1 mediated GC migration, invasion and EMT. **A** Transwell migration and invasion assays were performed to determine the role of ITGA5 in mediating the influence of AFAP1L1 on GC cells migratory and invasive ability. Scale bars, 50 μm. **B** Double fluorescent staining of cytoskeleton (red) and p-FAK (green) for indicated GC cells. Cytoskeleton was stained by rhodamine phalloidin and Nuclei was stained with DAPI (blue). Scale bars, 10 μm. **C** Adhesion assays to test cell-ECM adhesion capacity of indicated GC cells. BSA was used as negative control. **D** qRT-PCR analysis of ITGA5, E-cadherin and vimentin expression in AFAP1L1-interfered GC cells with ITGA5 further overexpression or knockdown. **E** Western blot analysis of ITGA5, E-cadherin and vimentin, FAK and ERK in AFAP1L1-interfered GC cells with ITGA5 further overexpression or knockdown. **P < 0.01; ***P < 0.001

To explore whether ITGA5 is necessary for AFAP1L1 mediated GC progression, we further overexpressed ITGA5 in AGS cells with AFAP1L1 downregulation and knocked down ITGA5 expression in MKN74 cells with AFAP1L1 overexpression. Transwell migration and invasion assays both showed ITGA5 overexpression in AGS^shAFAP1L1^ could restore its inhibited migratory and invasive ability, while knockdown of ITGA5 in MKN74^AFAP1L1^ suppressed its migration and invasion again (Fig. 6A). Previous studies reported that FAK plays important role in integrins mediated cytoskeleton remodeling, cell migration and adhesion [27]. Fluorescence staining of cytoskeleton and FAK simultaneously showed that ITGA5 could facilitate expression of activated FAK to cell edge and rearrange oval cytoskeleton of AGS^shAFAP1L1^ cells to fusiform type, while ITGA5 downregulation in MKN74^AFAP1L1^ cells caused contrary results (Fig. 6B). Adhesion assays revealed that AFAP1L1 promoted adhesion of GC cells to collagen, which could be interrupted by ITGA5 downregulation (Fig. 6C). To test whether ITGA5 participated in AFAP1L1 mediated EMT of GC cells, qRT-PCR and western blot results showed E-cadherin and vimentin expression in AGS^shAFAP1L1^ cells were recovered to levels in AGS^shCtrl^ cells after overexpression of ITGA5 and EMT markers expression in MKN74^AFAP1L1^ cells were recovered after knocking down of ITGA5 (Fig. 6D and E). Meantime, these experiments also confirmed FAK and ERK activation in AFAP1L1 overexpression cells was also mediated by ITGA5 (Fig. 6E). In brief, the role of AFAP1L1 in promoting GC progression is mediated by ITGA5.

### AFAP1L1 interacts with VAV2 and activates CDC42 signaling to promote ITGA5 expression

**Fig. 7:**
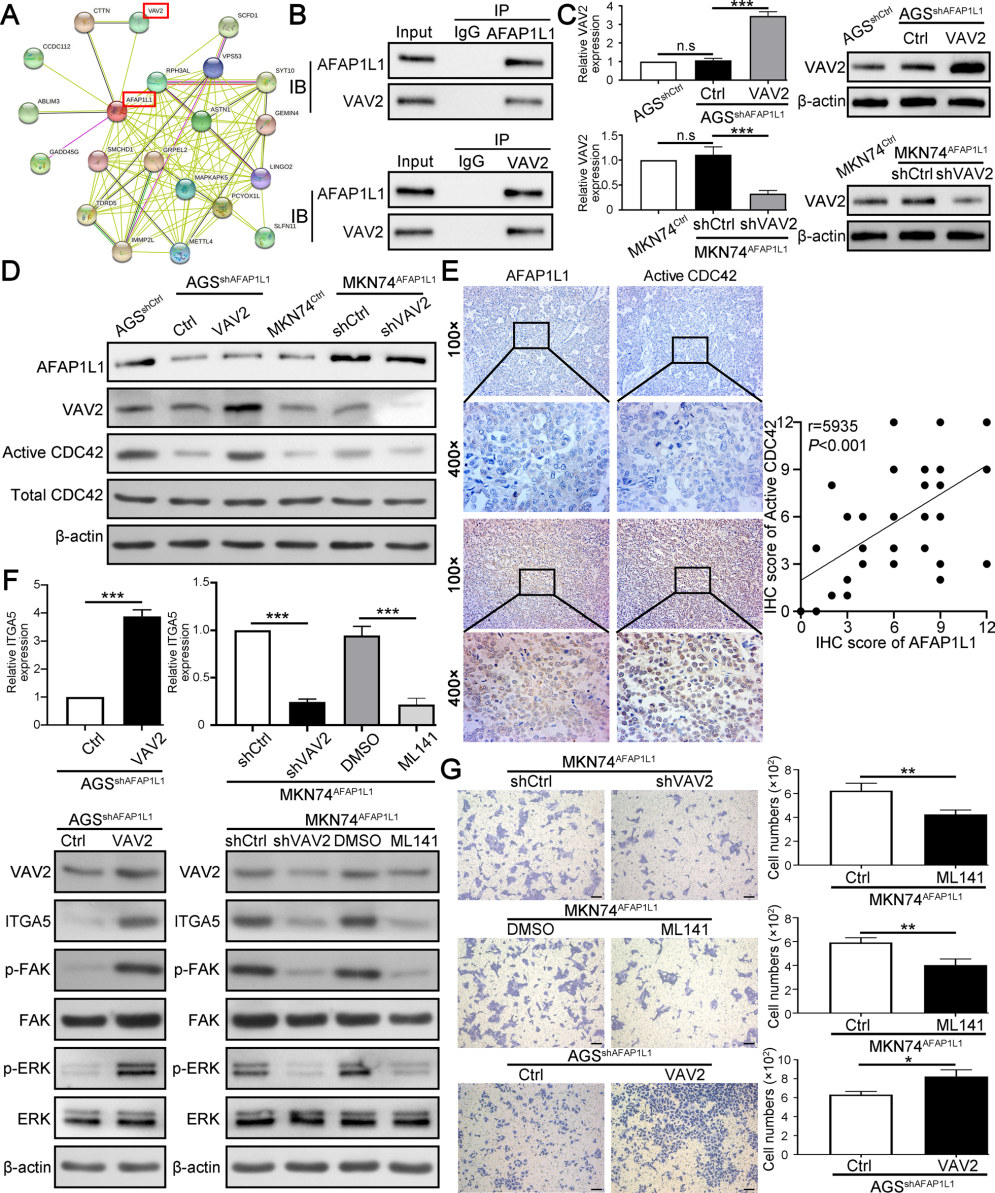
AFAP1L1 interacts with VAV2 and activates CDC42 signaling to promote ITGA5 expression. **A** The protein–protein interaction network of AFAP1L1 was constructed in STRING database (http://string-db.org). Among those interacted proteins, VAV2 attracted our attention. **B** Co-immunoprecipitation (Co-IP) analysis of the interaction between AFAP1L1 and VAV2 in AGS cells. **C** VAV2 knockdown and overexpression efficacy in indicated GC cells were determined by qRT-PCR and western blot. **D** Active CDC42 pull-down assays were performed to detect the level of active CDC42 in AFAP1L1-interfered GC cells with VAV2 further overexpression or knockdown. **E** The representative IHC images from serial sections of GC samples showed AFAP1L1 and active CDC42 expression in GC tissues with high or low AFAP1L1 expression. Scatter diagram of IHC staining scores showed the expression correlation between AFAP1L1 and active CDC42 in GC. Pearson correlation analysis was utilized to calculate correlation coefficient. **F** qRT-PCR analysis of ITGA5 and western blot analysis of ITGA5, FAK and ERK in AFAP1L1-interfered GC cells with VAV2 further overexpression or knockdown. ML141 (20 μm) was used to inhibit activation of CDC42 in MKN74^AFAP1L1^ cells. **G** Transwell invasion assays were performed to determine the role of VAV2 in mediating the influence of AFAP1L1 on GC cells invasive ability. ML141 (20 μm) was used to inhibit activation of CDC42 in MKN74^AFAP1L1^ cells. Scale bars, 50 μm. n.s, no significance; *P < 0.05; **P < 0.01; ***P < 0.001

Although the above results demonstrated AFAP1L1 promotes ITGA5 expression at transcriptional level, AFAP1L1 is a kind of adaptor protein without enzymatic activity and transcriptional activity. Therefore, we inferred that AFAP1L1 may interact with other effectors to affect ITGA5 mRNA expression. Previous studies had demonstrated AFAP1L1 could bind other proteins in different situation, such as vinculin [9], cortactin [28], Vav2 and Nck2 [29]. We first analyzed protein–protein interaction of AFAP1L1 in STRING database (www.string-db.org/) (Fig. 7A) and paid attention to VAV2 because of its role in Rho family GTPases activation [30]. CDC42 is a member of Rho family GTPases and could promote expression of ITGA5 at transcriptional level [31]. All the information made us to speculate AFAP1L1 may interact with VAV2 to activate CDC42. To verify this speculation, we performed co-immunoprecipitation assays and the results confirmed AFAP1L1 and VAV2 could interact with each other in GC cells (Fig. 7B). Then, we overexpressed VAV2 in AGS^shAFAP1L1^ cells and knocked down VAV2 expression in MKN74^AFAP1L1^ cells (Fig. 7C). AFAP1L1 downregulation in AGS inhibited expression level of active CDC42, while VAV2 overexpression in AGS^shAFAP1L1^ cells could rescue the active CDC42 expression level. The opposite results can be observed in MKN74^AFAP1L1^ cells after knocking down of VAV2 expression (Fig. 7D). In addition, manipulation of AFAP1L1 expression in GC cells had no significant influence on other Rho members, e.g. RhoA and Rac1 (Additional file 2: Fig. S5). IHC staining for AFAP1L1 and active CDC42 in serial sections of GC tissues showed AFAP1L1 was positively correlated with active CDC42 in GC tissues (Fig. 7E). Moreover, VAV2 overexpression in AGS^shAFAP1L1^ cells could also rescue the expression of ITGA5 and its downstream signaling FAK and ERK. VAV2 downregulation in MKN74^AFAP1L1^ cells inhibited ITGA5, FAK and ERK expression (Fig. 7F). ML141, a potent and selective inhibitor of CDC42 GTPase, could also inhibit ITGA5, FAK and ERK expression of MKN74^AFAP1L1^ cells (Fig. 7F). In addition, VAV2 overexpression restored the invasive capacity of AGS^shAFAP1L1^ cells, while VAV2 downregulation or ML141 treatment inhibited invasion of MKN74^AFAP1L1^ cells (Fig. 7G). In conclusion, AFAP1L1 promotes ITGA5 expression by interacting with VAV2 and activating CDC42 GTPases.

## Discussion

It’s well known that activation of oncogenes and/or inactivation of tumor suppressor genes are key events in tumor invasion-and-metastasis cascade. Present studies have shown that AFAP family members, including AFAP1/AFAP-110, AFAP1L1 and AFAP1L2/XB130, play vital role in many cellular functions, such as cell proliferation, migration, cytoskeletal reorganization, EMT, and so on [6, 8, 32]. To explore the role of AFAP family in GC, we first analyzed mRNA expression of AFAP family members in GC tissues using public database (TCGA, GEO). Interestingly, AFAP1L1 is the only one that is significantly upregulated in GC tissues, which is further verified using our GC samples. Besides, GC tissues with lymph node metastasis had significantly higher AFAP1L1 mRNA and protein expression than those without lymph node metastasis, indicating that AFAP1L1 may associate with GC metastasis. What’s more, in three members of AFAP family, AFAP1L1 is also the only one that associates with prognosis of GC patients and acts as an independent risk factor for overall survival of GC patients. From these studies and analyses, we can conclude that AFAP1L1 may participate in GC progression and could be served as a valuable prognostic biomarker.

AFAP1L1 gene was discovered when performing homology search of human genome using AFAP1 PH domain sequences [28]. Meanwhile, the initial study had found AFAP1L1 interacts with SH3 domain of cortactin and had the ability to induce podosome formation [28]. Subsequent studies demonstrated that AFAP1L1 localizes to invadopodia and intersects with several invadopodia pathway components to promote its formation in colorectal cancer and osteosarcoma [9, 29]. Many proteins that involve in invadopodia formation also participate in EMT, suggesting invadopodia formation could promote EMT [15]. Tumor cells occurring EMT have highly migratory and invasive ability by destroying epithelial cell-cell junction, modulating cell-matrix adhesion and promoting expression of ECM degrading proteinases [33]. In this study, we confirmed that AFAP1L1 could not only promote GC cells proliferation, migration, invasion in vitro but also tumor growth and metastasis in vivo. As an actin filament associated protein, AFAP1L1 remodels GC cells cytoskeleton to facilitate its movement. In addition, AFAP1L1 downregulates epithelial cell junction protein E-cadherin, epithelial cell adhesion molecules (EPCAM) and promotes expression of N-cadherin and vimentin, which is a significant marker for EMT occurrence. Therefore, AFAP1L1 could regulate cytoskeleton and EMT to affect invasion and metastasis of GC cells.

The integrin family comprises 24 transmembrane heterodimeric receptors generated by 18 α integrin and 8 β integrin subunits [23]. The extracellular domain of integrin receptors could bind specific peptide motifs of ECM proteins and its intracellular tail connects with cytoskeletal adaptor proteins and signaling molecules to regulate many signaling pathway, such as FAK, MAPK, PI3K/AKT, and YAP/TAZ signaling [34, 35]. Integrins signaling has been implicated in almost every step of tumor progression, including tumor proliferation, invasion, EMT and metastasis [23]. Jun-Fu Wang et al. had systematically analyzed expression and prognostic significance of integrin subunits in GC and concluded that ITGA5 may be used as a potential diagnostic marker for GC, indicating ITGA5 was the most important integrin subunit in GC [36]. GO enrichment analysis and KEGG analysis using TCGA STAD database both showed AFAP1L1 is directly or indirectly associated with integrin related signaling pathway. AFAP1L1 could promote ITGA5 expression and activate downstream FAK, ERK signaling pathway, which finally promotes migration, invasion and EMT of GC cells.

AFAP1L1 is adaptor protein without enzymatic activity and transcriptional activity. How AFAP1L1 regulates ITGA5 at transcriptional level is another interesting question to be solved. Among those proteins bound with AFAP1L1, VAV2 (Vav Guanine Nucleotide Exchange Factor 2) most likely attracted our attention. VAV2 protein functions as a guanine nucleotide exchange factor and scaffold protein to activate Rho/Rac family GTPases which play important role in cytoskeleton reorganization and tumor progression [37]. CDC42 is a member of the Rho GTPase family and acts as upstream protein of integrin signaling pathway. Many studies had reported CDC42 could promote ITGB1, ITGA5 expression at transcriptional level [31, 38, 39]. To pull all information together, we have reason to speculate AFAP1L1 may bind VAV2 to activate CDC42 and promote ITGA5 expression in GC cells. At last, a series of experiments proved our assumption and ML141, a CDC42 GTPase inhibitor, may be useful to prevent tumor progression for GC patients with high AFAP1L1 expression. Certainly, there are some limitations for this study. For example, how the interaction between AFA1P1L and VAV2 contributes to CDC42 activation and what mechanisms lead AFAP1L1 upregulation in GC are remaining subjects to be explored in the future.

## Conclusion

In summary, this study confirmed AFAP1L1 is the only AFAP family member that is significantly upregulated in GC and associated with poor prognosis of GC patients. AFAP1L1 could promote GC progression by facilitating proliferation, invasion, metastasis and EMT. Moreover, AFAP1L1 interacted with VAV2 to activate Rho GTPase CDC42 and further promote expression of ITGA5 and activation of downstream signaling pathway. AFAP1L1 could be used as a prognostic biomarker and potential therapeutic target for GC patients.

## Supplementary Information

**Additional file 1: Table S1.** The sequences of PCR primers used in this study. **Table S2.** The primary antibodies used in this study. **Table S3.** The association between AFAP1 expression and clinicopathological characteristics of GC patients. **Table S4.** The association between AFAP1L1 expression and clinicopathological characteristics of GC patients. **Table S5.** The association between AFAP1L2 expression and clinicopathological characteristics of GC patients. **Table S6.** Univariate and multivariate analysis of risk factors associated with overall survival of GC patients.

**Additional file 2: Fig. S1.** AFAP1L1 is significantly upregulated in GC tissues and cell lines. **Fig. S2.** High AFAP1L1 expression in GC promotes proliferation, invasion in vitro and growth, metastasis in vivo. **Fig. S3.** AFAP1L1 facilitates EMT process of GC cells. **Fig. S4.** AFAP1L1 is associated with integrin signaling and promotes ITGA5 expression in GC. **Fig. S5.** Western blot analysis of total RhoA, total Rac1 and their active forms in AGS^shAFAP1L1^, MKN74^AFAP1L1^ and their control cells.

## Data Availability

The datasets used and/or analyzed during the current study available from the corresponding author on reasonable request. All data generated or analyzed during this study are included in this published article.

## Abbreviations

GC: Gastric cancer
AFAP1L1: Actin filament associated protein 1 like 1
EMT: Epithelial-to-mesenchymal transition
ITGA5: Integrin subunit alpha 5
VAV2: VAV guanine nucleotide exchange factor 2
CDC42: Cell division cycle 42
LNM: Lymph node metastasis
ANGT: Adjacent nontumorous gastric tissues
TCGA: The Cancer Genome Atlas
GSEA: Gene Set Enrichment Analysis
KEGG: Kyoto Encyclopedia of Genes and Genomes
GEO: Gene Expression Omnibus
co-IP: Co-immunoprecipitation
DEG: Differentially expressed gene
