# Supplementary material for: AFAP1L1 promotes gastric cancer progression by interacting with VAV2 to facilitate CDC42-mediated activation of ITGA5 signaling pathway
Source: J Transl Med. 2023 Jan 11;21:18. doi: 10.1186/s12967-023-03871-8 (PMC9835296; doi:10.1186/s12967-023-03871-8)
Supplement: Supplementary file 1 — Additional file 1: Table S1. The sequences of PCR primers used in this study. Table S2. The primary antibodies used in this study. Table S3. The association between AFAP1 expression and clinicopathological characteristics of GC patients. Table S4. The association between AFAP1L1 expression and clinicopathological characteristics of GC patients. Table S5. The association between AFAP1L2 expression and clinicopathological characteristics of GC patients. Table S6. Univariate and multivariate analysis of risk factors associated with overall survival of GC patients. [file 12967_2023_3871_MOESM1_ESM.docx]

**Table S1. The sequences of PCR primers used in this study**

| **Genes** | **Sequence (5’→3’)** |
| --- | --- |
| AFAP1 | F: AGCAGCTCTTATGAGTCGTATGA |
|  | R: GCACAGCAGTTTGGTGTCTTT |
| AFAP1L1 | F: CGAGTACCTCAGCGATACCAC |
|  | R: CTTCAAAGAGGGATTCCACGAA |
| AFAP1L2 | F: ACAGCTTGCCATCCCAAAGAC |
|  | R: CAGCCTCTCCGTCCTCATTG |
| GAPDH | F: ACAACTTTGGTATCGTGGAAGG |
|  | R: GCCATCACGCCACAGTTTC |
| E-cadherin | F: CGAGAGCTACACGTTCACGG |
|  | R: GGGTGTCGAGGGAAAAATAGG |
| EPCAM | F: TGATCCTGACTGCGATGAGAG |
|  | R: CTTGTCTGTTCTTCTGACCCC |
| Vimentin | F: AGTCCACTGAGTACCGGAGAC |
|  | R: CATTTCACGCATCTGGCGTTC |
| N-cadherin | F: TGCGGTACAGTGTAACTGGG |
|  | R: GAAACCGGGCTATCTGCTCG |
| ITGA5 | F: GCCTGTGGAGTACAAGTCCTT |
|  | R: AATTCGGGTGAAGTTATCTGTGG |
| ITGB3 | F: GTGACCTGAAGGAGAATCTGC |
|  | R: CCGGAGTGCAATCCTCTGG |
| VAV2 | F: CTGTTTGACCCCTTTGACCTC |
|  | R: GACGCAGTCGTAGATGTCCTC |

**Table S2. The primary antibodies used in this study**

| **Primary Antibodies** | **Origin** | **Application** | **Dilution** |
| --- | --- | --- | --- |
| AFAP1L1 | Santa Cruz (sc-376700) | WB | 1:500 |
|  |  | IF | 1:200 |
| AFAP1L1 | GeneTex (GTX32040) | IHC | 1:100 |
| β-actin | ZSGB-BIO (TA-09) | WB | 1:2000 |
| E-cadherin | CST (#3195) | WB | 1:1000 |
|  |  | IHC | 1:400 |
|  |  | IF | 1:200 |
| Vimentin | Proteintech (60330-1-Ig) | WB | 1:1000 |
|  |  | IHC | 1:200 |
|  |  | IF | 1:100 |
| EPCAM | CST (#2929) | WB | 1:500 |
| N-cadherin | GeneTex (GTX82992) | WB | 1:1000 |
| ITGB3 | Proteintech (18309-1-AP) | WB | 1:1000 |
| ITGA5 | Proteintech (10569-1-AP) | WB | 1:1000 |
|  |  | IHC | 1:200 |
| p-FAK | Affinity (AF3398) | WB | 1:1000 |
|  |  | IHC | 1:200 |
|  |  | IF | 1:100 |
| FAK | Affinity (AF6397) | WB | 1:1000 |
| p-ERK | CST (#4376) | WB | 1:1000 |
|  |  | IHC | 1:200 |
| ERK | CST (#4696) | WB | 1:1000 |
| VAV2 | Proteintech (21924-1-AP) | WB | 1:500 |
| Active CDC42 | NewEast Bio (#26905) | WB | 1:500 |

**Table S3. The association between AFAP1 expression and clinicopathological characteristics of GC patients**

| **Characteristic** | **Low expression of AFAP1** | **High expression of AFAP1** | ***p*** |
| --- | --- | --- | --- |
| n | 187 | 188 |  |
| **T stage, n (%)** |  |  | 0.149 |
| T1 | 13 (3.5%) | 6 (1.6%) |  |
| T2 | 44 (12%) | 36 (9.8%) |  |
| T3 | 84 (22.9%) | 84 (22.9%) |  |
| T4 | 43 (11.7%) | 57 (15.5%) |  |
| **N stage, n (%)** |  |  | 0.215 |
| N0 | 58 (16.2%) | 53 (14.8%) |  |
| N1 | 49 (13.7%) | 48 (13.4%) |  |
| N2 | 43 (12%) | 32 (9%) |  |
| N3 | 30 (8.4%) | 44 (12.3%) |  |
| **M stage, n (%)** |  |  | 0.777 |
| M0 | 168 (47.3%) | 162 (45.6%) |  |
| M1 | 14 (3.9%) | 11 (3.1%) |  |
| **Pathologic stage, n (%)** |  |  | 0.291 |
| Stage I | 31 (8.8%) | 22 (6.2%) |  |
| Stage II | 57 (16.2%) | 54 (15.3%) |  |
| Stage III | 67 (19%) | 83 (23.6%) |  |
| Stage IV | 21 (6%) | 17 (4.8%) |  |
| **Gender, n (%)** |  |  | 0.718 |
| Female | 69 (18.4%) | 65 (17.3%) |  |
| Male | 118 (31.5%) | 123 (32.8%) |  |
| **Age, n (%)** |  |  | 0.861 |
| <=65 | 84 (22.6%) | 80 (21.6%) |  |
| >65 | 103 (27.8%) | 104 (28%) |  |
| **Histological type, n (%)** |  |  | **0.024** |
| Diffuse Type | 30 (8%) | 33 (8.8%) |  |
| Mucinous Type | 3 (0.8%) | 16 (4.3%) |  |
| Not Otherwise Specified | 111 (29.7%) | 96 (25.7%) |  |
| Papillary Type | 3 (0.8%) | 2 (0.5%) |  |
| Signet Ring Type | 3 (0.8%) | 8 (2.1%) |  |
| Tubular Type | 37 (9.9%) | 32 (8.6%) |  |
| **Histologic grade, n (%)** |  |  | 0.265 |
| G1 | 4 (1.1%) | 6 (1.6%) |  |
| G2 | 76 (20.8%) | 61 (16.7%) |  |
| G3 | 104 (28.4%) | 115 (31.4%) |  |
| **Anatomic neoplasm subdivision, n (%)** |  |  | **0.014** |
| Antrum/Distal | 71 (19.7%) | 67 (18.6%) |  |
| Cardia/Proximal | 30 (8.3%) | 18 (5%) |  |
| Fundus/Body | 52 (14.4%) | 78 (21.6%) |  |
| Gastroesophageal Junction | 22 (6.1%) | 19 (5.3%) |  |
| Other | 4 (1.1%) | 0 (0%) |  |

**Table S4. The association between AFAP1L1 expression and clinicopathological characteristics of GC patients**

| **Characteristic** | **Low expression of AFAP1L1** | **High expression of AFAP1L1** | ***p*** |
| --- | --- | --- | --- |
| n | 187 | 188 |  |
| **T stage, n (%)** |  |  | **0.014** |
| T1 | 14 (3.8%) | 5 (1.4%) |  |
| T2 | 47 (12.8%) | 33 (9%) |  |
| T3 | 84 (22.9%) | 84 (22.9%) |  |
| T4 | 40 (10.9%) | 60 (16.3%) |  |
| **N stage, n (%)** |  |  | 0.768 |
| N0 | 59 (16.5%) | 52 (14.6%) |  |
| N1 | 50 (14%) | 47 (13.2%) |  |
| N2 | 36 (10.1%) | 39 (10.9%) |  |
| N3 | 34 (9.5%) | 40 (11.2%) |  |
| **M stage, n (%)** |  |  | 0.965 |
| M0 | 167 (47%) | 163 (45.9%) |  |
| M1 | 12 (3.4%) | 13 (3.7%) |  |
| **Pathologic stage, n (%)** |  |  | 0.114 |
| Stage I | 34 (9.7%) | 19 (5.4%) |  |
| Stage II | 54 (15.3%) | 57 (16.2%) |  |
| Stage III | 68 (19.3%) | 82 (23.3%) |  |
| Stage IV | 17 (4.8%) | 21 (6%) |  |
| **Gender, n (%)** |  |  | 0.884 |
| Female | 68 (18.1%) | 66 (17.6%) |  |
| Male | 119 (31.7%) | 122 (32.5%) |  |
| **Age, n (%)** |  |  | 1.000 |
| <=65 | 82 (22.1%) | 82 (22.1%) |  |
| >65 | 104 (28%) | 103 (27.8%) |  |
| **Histological type, n (%)** |  |  | **0.024** |
| Diffuse Type | 27 (7.2%) | 36 (9.6%) |  |
| Mucinous Type | 5 (1.3%) | 14 (3.7%) |  |
| Not Otherwise Specified | 111 (29.7%) | 96 (25.7%) |  |
| Papillary Type | 0 (0%) | 5 (1.3%) |  |
| Signet Ring Type | 5 (1.3%) | 6 (1.6%) |  |
| Tubular Type | 39 (10.4%) | 30 (8%) |  |
| **Histologic grade, n (%)** |  |  | 0.060 |
| G1 | 4 (1.1%) | 6 (1.6%) |  |
| G2 | 80 (21.9%) | 57 (15.6%) |  |
| G3 | 101 (27.6%) | 118 (32.2%) |  |
| **Anatomic neoplasm subdivision, n (%)** |  |  | 0.195 |
| Antrum/Distal | 72 (19.9%) | 66 (18.3%) |  |
| Cardia/Proximal | 22 (6.1%) | 26 (7.2%) |  |
| Fundus/Body | 58 (16.1%) | 72 (19.9%) |  |
| Gastroesophageal Junction | 26 (7.2%) | 15 (4.2%) |  |
| Other | 3 (0.8%) | 1 (0.3%) |  |

**Table S5. The association between AFAP1L2 expression and clinicopathological characteristics of GC patients**

| **Characteristic** | **Low expression of AFAP1L2** | **High expression of AFAP1L2** | ***p*** |
| --- | --- | --- | --- |
| n | 187 | 188 |  |
| **T stage, n (%)** |  |  | 0.564 |
| T1 | 10 (2.7%) | 9 (2.5%) |  |
| T2 | 46 (12.5%) | 34 (9.3%) |  |
| T3 | 82 (22.3%) | 86 (23.4%) |  |
| T4 | 48 (13.1%) | 52 (14.2%) |  |
| **N stage, n (%)** |  |  | 0.906 |
| N0 | 55 (15.4%) | 56 (15.7%) |  |
| N1 | 52 (14.6%) | 45 (12.6%) |  |
| N2 | 37 (10.4%) | 38 (10.6%) |  |
| N3 | 36 (10.1%) | 38 (10.6%) |  |
| **M stage, n (%)** |  |  | 0.253 |
| M0 | 165 (46.5%) | 165 (46.5%) |  |
| M1 | 9 (2.5%) | 16 (4.5%) |  |
| **Pathologic stage, n (%)** |  |  | 0.108 |
| Stage I | 29 (8.2%) | 24 (6.8%) |  |
| Stage II | 58 (16.5%) | 53 (15.1%) |  |
| Stage III | 78 (22.2%) | 72 (20.5%) |  |
| Stage IV | 12 (3.4%) | 26 (7.4%) |  |
| **Gender, n (%)** |  |  | 0.474 |
| Female | 63 (16.8%) | 71 (18.9%) |  |
| Male | 124 (33.1%) | 117 (31.2%) |  |
| **Age, n (%)** |  |  | 0.953 |
| <=65 | 83 (22.4%) | 81 (21.8%) |  |
| >65 | 103 (27.8%) | 104 (28%) |  |
| **Histological type, n (%)** |  |  | 0.052 |
| Diffuse Type | 32 (8.6%) | 31 (8.3%) |  |
| Mucinous Type | 3 (0.8%) | 16 (4.3%) |  |
| Not Otherwise Specified | 105 (28.1%) | 102 (27.3%) |  |
| Papillary Type | 4 (1.1%) | 1 (0.3%) |  |
| Signet Ring Type | 6 (1.6%) | 5 (1.3%) |  |
| Tubular Type | 36 (9.6%) | 33 (8.8%) |  |
| **Histologic grade, n (%)** |  |  | 0.169 |
| G1 | 2 (0.5%) | 8 (2.2%) |  |
| G2 | 71 (19.4%) | 66 (18%) |  |
| G3 | 111 (30.3%) | 108 (29.5%) |  |
| **Anatomic neoplasm subdivision, n (%)** |  |  | 0.385 |
| Antrum/Distal | 61 (16.9%) | 77 (21.3%) |  |
| Cardia/Proximal | 26 (7.2%) | 22 (6.1%) |  |
| Fundus/Body | 69 (19.1%) | 61 (16.9%) |  |
| Gastroesophageal Junction | 18 (5%) | 23 (6.4%) |  |
| Other | 3 (0.8%) | 1 (0.3%) |  |

**Table S6. Univariate and multivariate analysis of risk factors associated with overall survival of GC patients**

| **Characteristics** | **Total (N)** | **Univariate analysis** | |  | **Multivariate analysis** | |
| --- | --- | --- | --- | --- | --- | --- |
|  |  | **Hazard ratio (95% CI)** | ***P*** |  | **Hazard ratio (95% CI)** | ***P*** |
| **T stage** | 362 |  |  |  |  |  |
| T1 | 18 | Reference |  |  |  |  |
| T2&T3&T4 | 344 | 8.829(1.234-63.151) | **0.030** |  | 4.951(0.677-36.214) | 0.115 |
| **N stage** | 352 |  |  |  |  |  |
| N0 | 107 | Reference |  |  |  |  |
| N1&N2&N3 | 245 | 1.925(1.264-2.931) | **0.002** |  | 1.450(0.809-2.598) | 0.212 |
| **M stage** | 352 |  |  |  |  |  |
| M0 | 327 | Reference |  |  |  |  |
| M1 | 25 | 2.254(1.295-3.924) | **0.004** |  | 2.127(1.154-3.922) | **0.016** |
| **Pathologic stage** | 347 |  |  |  |  |  |
| Stage I&Stage II | 160 | Reference |  |  |  |  |
| Stage III&Stage IV | 187 | 1.947(1.358-2.793) | **<0.001** |  | 1.251(0.754-2.075) | 0.386 |
| **Gender** | 370 |  |  |  |  |  |
| Female | 133 | Reference |  |  |  |  |
| Male | 237 | 1.267(0.891-1.804) | 0.188 |  |  |  |
| **Histologic grade** | 361 |  |  |  |  |  |
| G1 | 10 | Reference |  |  |  |  |
| G2&G3 | 351 | 1.957(0.484-7.910) | 0.346 |  |  |  |
| **Age** | 367 |  |  |  |  |  |
| <=65 | 163 | Reference |  |  |  |  |
| >65 | 204 | 1.620(1.154-2.276) | **0.005** |  | 1.796(1.240-2.601) | **0.002** |
| **Anatomic neoplasm subdivision** | 357 |  |  |  |  |  |
| Gastroesophageal Junction&Antrum/Distal | 177 | Reference |  |  |  |  |
| Cardia/Proximal&Fundus/Body&Other | 180 | 1.046(0.747-1.465) | 0.792 |  |  |  |
| **Histological type** | 369 |  |  |  |  |  |
| Not Otherwise Specified&Tubular Type | 271 | Reference |  |  |  |  |
| Signet Ring Type&Papillary Type&Mucinous Type&Diffuse Type | 98 | 0.868(0.600-1.256) | 0.452 |  |  |  |
| **AFAP1** | 370 |  |  |  |  |  |
| Low | 184 | Reference |  |  |  |  |
| High | 186 | 1.273(0.916-1.771) | 0.151 |  |  |  |
| **AFAP1L1** | 370 |  |  |  |  |  |
| Low | 185 | Reference |  |  |  |  |
| High | 185 | 1.560(1.116-2.181) | **0.009** |  | 1.471(1.029-2.104) | **0.034** |
| **AFAP1L2** | 370 |  |  |  |  |  |
| Low | 183 | Reference |  |  |  |  |
| High | 187 | 0.864(0.622-1.200) | 0.382 |  |  |  |
