# Supplementary material for: AFAP1L1 promotes gastric cancer progression by interacting with VAV2 to facilitate CDC42-mediated activation of ITGA5 signaling pathway
Source: J Transl Med. 2023 Jan 11;21:18. doi: 10.1186/s12967-023-03871-8 (PMC9835296; doi:10.1186/s12967-023-03871-8)
Supplement: Supplementary file 2 — Additional file 2: Fig. S1. AFAP1L1 is significantly upregulated in GC tissues and cell lines. Fig. S2. High AFAP1L1 expression in GC promotes proliferation, invasion in vitro and growth, metastasis in vivo. Fig. S3. AFAP1L1 facilitates EMT process of GC cells. Fig. S4. AFAP1L1 is associated with integrin signaling and promotes ITGA5 expression in GC. Fig. S5. Western blot analysis of total RhoA, total Rac1 and their active forms in AGSshAFAP1L1, MKN74AFAP1L1 and their control cells. [file 12967_2023_3871_MOESM2_ESM.docx]

**
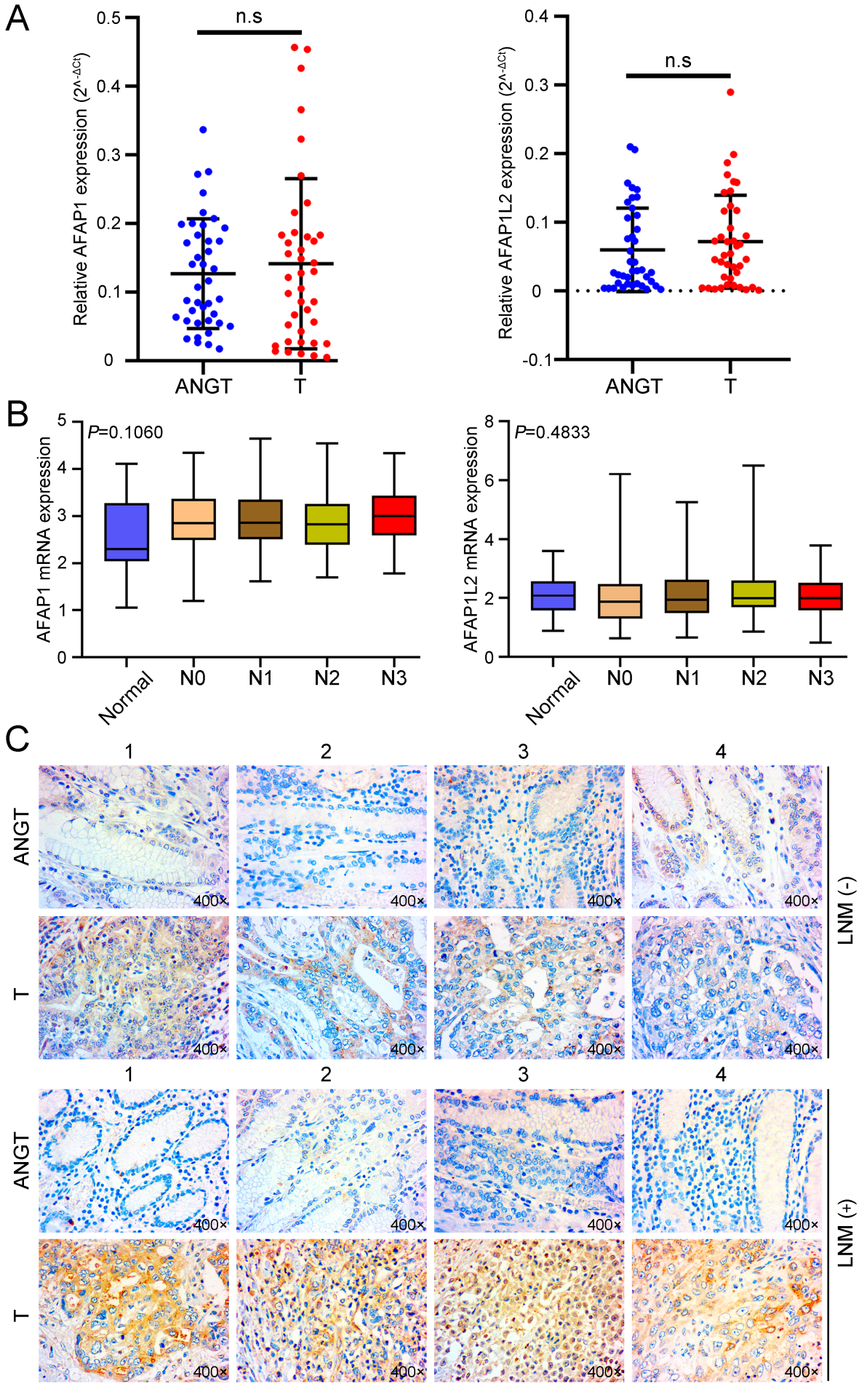
**

**Fig. S1 AFAP1L1 is significantly upregulated in GC tissues and cell lines.** (A) qRT-PCR analysis of AFAP1 and AFAP1L2 mRNA expression in 40 pairs of randomly selected snap-frozen GC tissues and ANGTs. (B) The expression of AFAP1 and AFAP1L2 in TCGA STAD database stratified by the number of lymph node metastasis. (C) IHC images confirmed the expression of AFAP1L1 in GC tissues matched in Fig. 1D. Abbreviation: ANGT, adjacent nontumorous gastric tissue; T, gastric cancer tissue. n.s, no significance.


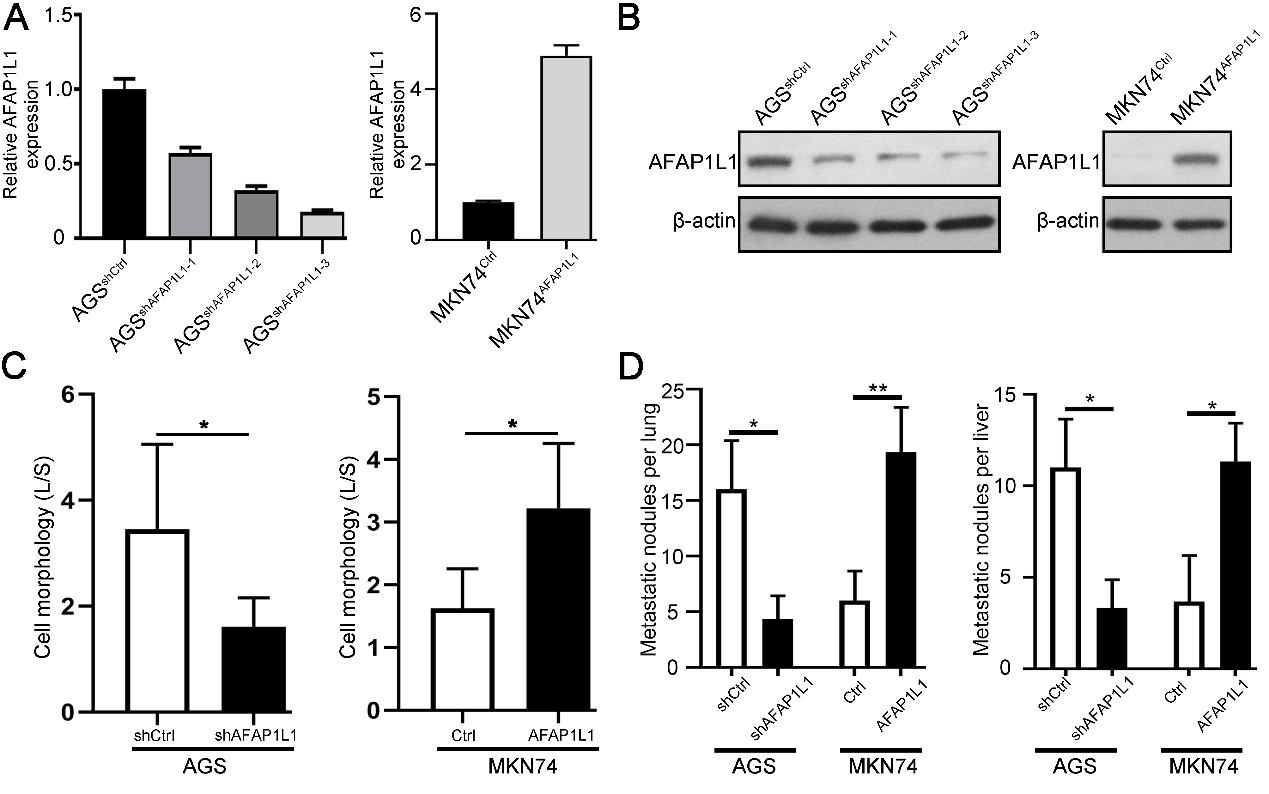


**Fig. S2 High AFAP1L1 expression in GC promotes proliferation, invasion *in vitro* and growth, metastasis *in vivo*.** (A-B) qRT-PCR (A) and western blot (B) were performed to determine AFAP1L1 knockdown efficacy in AGS cells and AFAP1L1 overexpression efficacy in MKN74 cells. (C) The cell morphology in Fig. 3D was calculated and compared by the long diameter (L) divided by the short diameter (S) of cytoskeleton. (D) The number of metastatic nodules per lung and liver was calculated and compared between AGS^shAFAP1L1^, MKN74^AFAP1L1^ and their control cells. *, *P*<0.05; **, *P*<0.01.


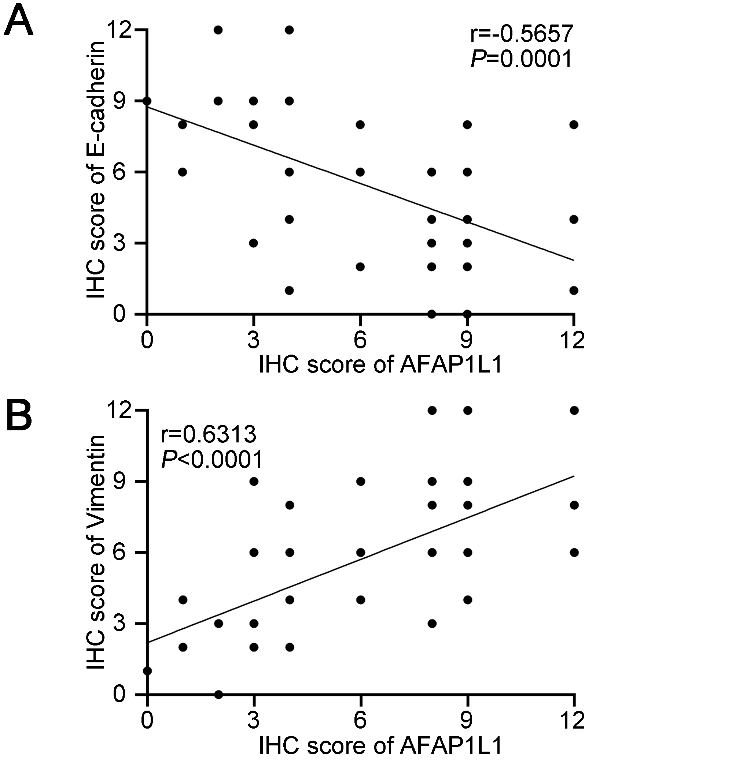


**Fig. S3 AFAP1L1 facilitates EMT process of GC cells.** (A) Scatter diagram of IHC staining scores showed the expression correlation between AFAP1L1 and E-cadherin in GC. (B) Scatter diagram of IHC staining scores showed the expression correlation between AFAP1L1 and vimentin in GC. Pearson correlation analysis was utilized to calculate correlation coefficient.


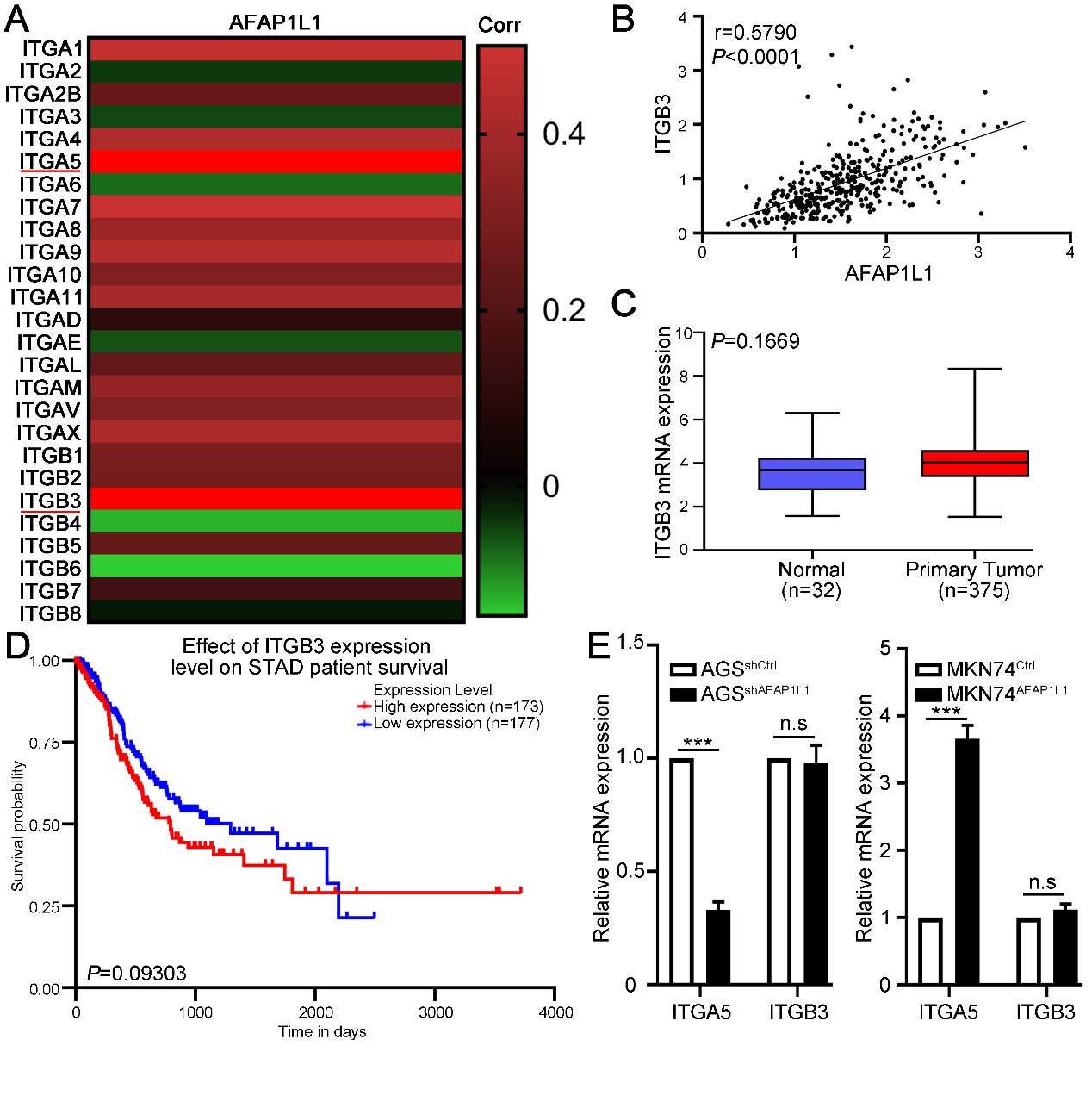


**Fig. S4 AFAP1L1 is associated with integrin signaling and promotes ITGA5 expression in GC.** (A) The correlation heatmap (correlation coefficient) analyzed using TCGA STAD database showed the correlation between AFAP1L1 and integrin family members. Among the 26 members, ITGA5 and ITGB3 had the largest correlation coefficient. (B) Pearson correlation analysis of AFAP1L1 and ITGA5 in GC using data from TCGA STAD database. (C) Analysis using TCGA STAD database showed ITGA5 expression in GC tissues and normal gastric tissues. (D) Analysis using TCGA STAD database showed the effect of ITGA5 expression on prognosis of GC patients. (E) qRT-PCR analysis to detect the effect of AFAP1L1 overexpression or knockdown on the expression of ITGA5, ITGB3.


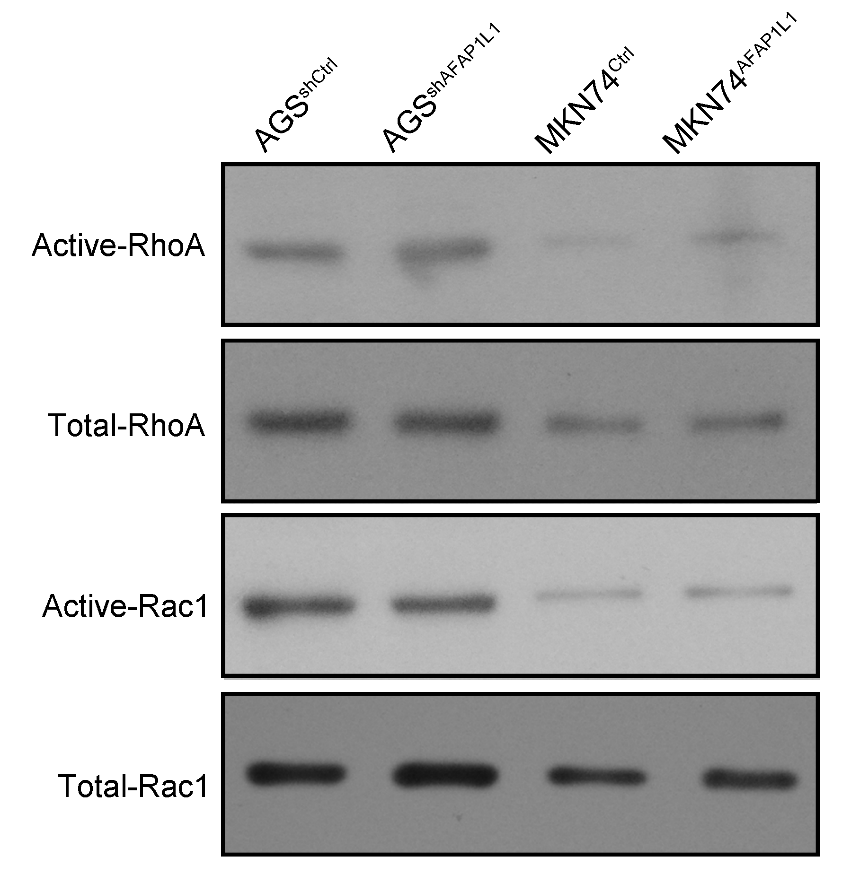


**Fig. S5** Western blot analysis of total RhoA, total Rac1 and their active forms in AGS^shAFAP1L1^, MKN74^AFAP1L1^ and their control cells.
